# Association of birth weight with lung function and the mediating role of gut microbiota: A STROBE 2-step Mendelian randomization study

**DOI:** 10.1097/MD.0000000000046294

**Published:** 2025-12-26

**Authors:** Limin Cao, Yaochen Zhang, Yun Li, Qiwei Wang, Keyi Fan, Xinyue Zhang, Yahui Wen, Zhenglong Li, Xinhua Zhang

**Affiliations:** a Department of Pediatrics, Shanxi Medical University, Taiyuan, China; b Department of Neonatology, Shanxi Medical University Affiliated Children’s Hospital, Shanxi Children’s Hospital (Shanxi Maternal and Child Health Hospital), Taiyuan, China; c Shanxi Medical University, Taiyuan, China.

**Keywords:** birth weight, gut microbiota, lung, Mendelian randomization, pulmonology

## Abstract

Accumulating evidence has suggested that low birth weight (LBW) influences lung function deficit in adulthood. The aim of this study was to examine the relationship between LBW and adult lung function deficit and to explore the potential mediating effect of the gut microbiota in this relationship. Using summary data from genome-wide association studies, we applied 2-sample Mendelian randomization to investigate the association between birth weight and adult lung function deficit. Various MR analysis methods were used, including the inverse variance weighted, MR-Egger, weighted median, simple mode and weighted mode. Furthermore, a mediation analysis was performed to identify the potential mediating role of 412 known bacterial microbiota. This study identified significant genetic associations between birth weight and forced vital capacity, forced expiratory volume in 1-s (FEV1), FEV1/forced vital capacity ratio, and lung volume. In addition, a 2-step MR analysis indicated that the effect of LBW on lung function was mediated by specific gut microbiota, including g_Blautia, s_Subdoligranulum_unclassified, and s_Ruminococcus_obeum. LBW is associated with adult lung function deficit, with gut microbiota partially mediating this relationship. These findings provide important insights for the prevention of lung function impairment in individuals with LBW.

## 1. Introduction

Low birth weight (LBW) imposes a significant public health burden, affecting approximately 14.7% of live births worldwide.^[1]^ LBW is associated with higher mortality rates in children and adolescents.^[2]^ Furthermore, the developmental origins of health and disease (DOHaD) paradigm indicates that LBW could be linked to adverse lifelong health outcomes, including gastrointestinal, respiratory, and metabolic diseases.^[3,4]^ Although the prevalence of LBW is gradually decreasing at a rate of about 1.0% per year due to advances in prenatal screening, it continues to have a negative impact on human health.^[5]^

Adult lung function deficit is associated not only with respiratory complications, but also with all-cause mortality and cardiovascular disease.^[6]^ Currently, we measure adult lung function deficit using indicators such as forced vital capacity (FVC), FEV1, FEV1/FVC ratio and lung volume. Pulmonary development begins in utero and continues into late adolescence and early adulthood. Consequently, as an indicator of intrauterine growth restriction, LBW is associated with lung function outcomes.^[7–12]^

The mechanisms linking LBW to adult lung function deficits remain unclear. Elucidating the pathways between LBW and impaired lung function could inform clinical and public health interventions. We hypothesize that the gut microbiota may serve as a potential mediator in the association between LBW and lung function impairment, although the mediation effect has not been previously studied. The gastrointestinal tract harbors a complex and highly diverse microbial ecosystem that plays a critical role in maintaining host health and regulating various physiological processes.^[13]^ The gut microbiota is primarily composed of 4 major phyla: Firmicutes, Bacteroidetes, Proteobacteria and Actinobacteria. Recent studies have demonstrated the LBW is associated with gut microbiota imbalance.^[14]^ Meanwhile, the gut microbiota has a profound influence on systemic inflammation and chronic disease.^[15,16]^ The existence of the gut–lung axis, a vital cross-talk between gut microbiota and the lungs, could have significant implications for disease etiology and therapeutic strategies.^[17]^

Mendelian randomization (MR) is an epidemiological approach employed to infer causal relationships between exposures and outcomes.^[18]^ Single nucleotide polymorphisms (SNPs) identified by genome-wide association studies (GWAS) serve as instrumental variables (IVs). MR can mitigate the bias from unmeasured confounders by leveraging Mendelian inheritance patterns to avoid reverse causation.^[19]^ In this study, we performed a 2-step MR analysis to determine the genetic causal relationship between LBW and lung function, and to explore the potential mediating role of the gut microbiota.

## 2. Methods

### 2.1. Study design

**Figure 1.**
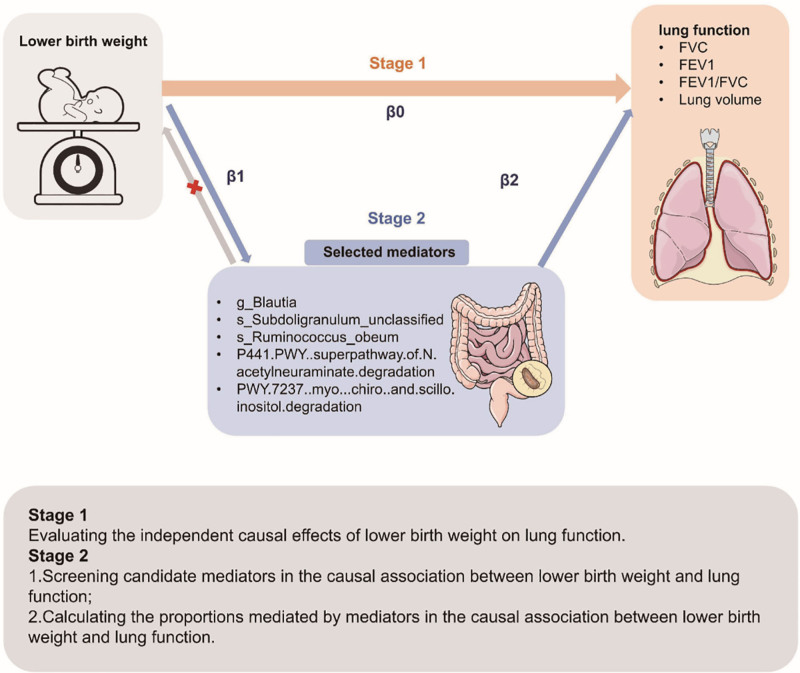
Overview of the MR study design. FEV1 = forced expiratory volume in 1-s, FVC = forced vital capacity forced.

In this study, a 2-step MR design was used to explore the causal mediating effect of gut microbiota (mediator) on the relationship between birth weight (exposure) and lung function (outcome) (Fig. S1, Supplemental Digital Content, https://links.lww.com/MD/Q813). First, we identified the birth weight significantly affecting lung function (β0), including FVC, FEV1, FEV1/FVC and lung volume. Subsequently, we evaluated the effect of potential mediating using a 2-step MR, which consisted of 2 steps: the first examined birth weight’s causal effect on gut microbiota (β1), while the second examined gut microbiota causal effect on lung function (β2). The indirect effect (mediated effect) was obtained by subtracting the direct effect from the total effect^[20]^ (Fig. 1).

Ethics committee approval and informed patient consent was provided for the original study. According to the guidance received from our research ethics board, no ethics review is required for studies that use public datasets.

### 2.2. Data sources

The birth weight GWAS dataset obtained from UKB contained 261,932 samples and 9851,867 SNPs (Table S1, Supplemental Digital Content, https://links.lww.com/MD/Q813). The GWAS summary data for 412 gut microbiota species were acquired, comprising 7738 individuals of European descent.^[21]^ Genetic variants associated with FVC and FEV1 were extracted from the GWAS to date conducted by the MRC-IEU, with 421,986 samples and 9851,867 SNPs. Additionally, genetic variants associated with FEV1/FVC and lung volume with a total of 51,396 and 32,860 Europeans, separately^[22]^ (Table 1).

**Table 1:** Summary of the GWAS data used in the MR analyses.

	GWAS ID	Trait	Year	Population	Sample Size	Number of SNPs	Author
Exposure	ukb-b-13378	Birth weight	2018	European	261,932	9,851,867	Ben Elsworth
Mediator	ebi-a-GCST90027446-ebi-a-GCST90027857	412 gut microbiota	2022	European	7738	5,563,857	Lopera-Maya EA
Outcome	ukb-b-7953	FVC	2018	European	421,986	9,851,867	Ben Elsworth
Outcome	ukb-b-19657	FEV1	2018	European	421,986	9,851,867	Ben Elsworth
Outcome	ieu-b-4856	FEV1/FVC	2022	European	51,396	9,703,538	Howe LJ
Outcome	ebi-a-GCST90016668	Lung volume	2021	European	32,860	9,275,407	Liu Y

FEV1 = forced expiratory volume in 1-s, FVC = forced vital capacity forced, MR = Mendelian randomization.

### 2.3. Instrumental variables

The IVs should satisfy the following 3 assumptions: the IVs are associated with the exposure (Assumption 1); the IVs are not linked with any confounders (Assumption 2); and the IVs influence the outcome only through the exposure (Assumption 3). We set a genome-wide significance threshold of 5 × 10^−8^ for each GWAS. Moreover, SNPs without linkage disequilibrium (*r*^2^ <0.01 and clump distance >10,000 kb) were used as instrument variables to avoid any bias.^[23]^ *F*-statistic was calculated to confirm the strength of IVs, discarding those with *F* <10 to prevent weak IVs.

### 2.4. Mendelian randomization analyses

For the statistical study, inverse variance weighted (IVW), MR-Egger, weighted median, simple mode, weighted mode were used. Among them, IVW was the main statistical method. We assessed heterogeneity using the Cochran Q statistic and conducted horizontal pleiotropy by MR-Egger regression intercept. Leave-one-out analysis was performed to determine whether the results were driven by a specific SNP. All MR analyses were performed using the R package TwoSampleMR v0.6.2.

## 3. Results

### 3.1. Effect of birth weight on lung function

**Figure 2.**
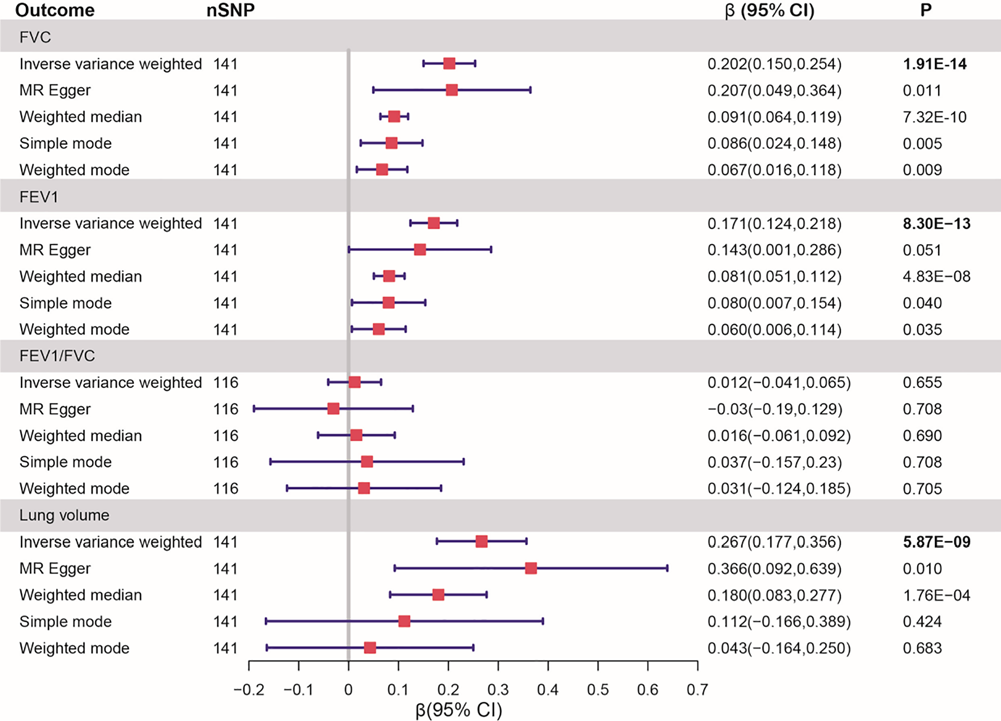
Univariable Mendelian randomization estimates of the causal association of birth weight with lung function. FEV1 = forced expiratory volume in 1-s, FVC = forced vital capacity forced.

In IVW study, birth weight showed significant associations with lung function, including FVC (β: 0.202), FEV1 (β: 0171) and lung volume (β: 0.267) (Fig. 2). In addition, there was no statistically significant effect of the LBW on FEV1/FVC (Table S2, Supplemental Digital Content, https://links.lww.com/MD/Q813).

### 3.2. Effect of birth weight on 412 gut microbiota

**Figure 3.**
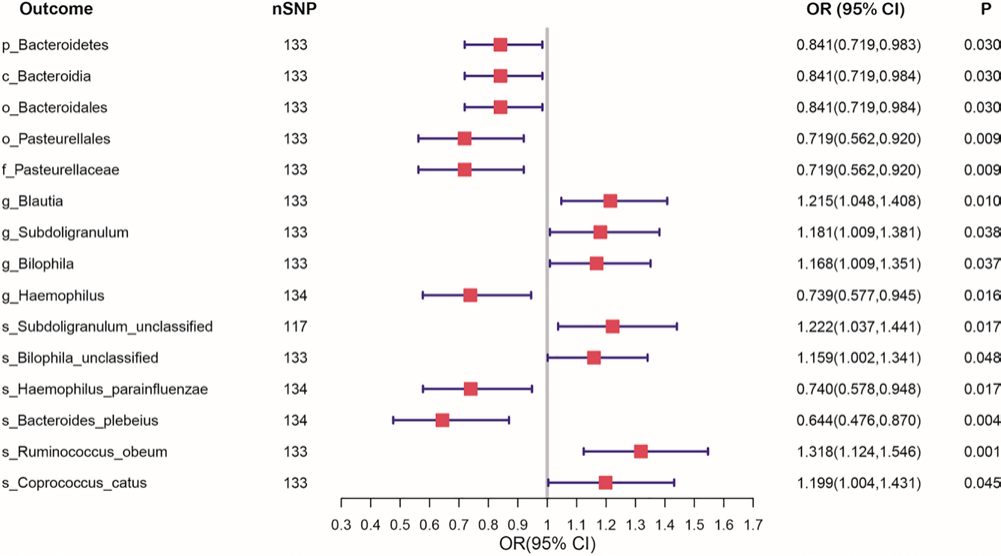
Univariable Mendelian randomization estimates of the causal association of each birth weight with gut microbiota abundance. FEV1 = forced expiratory volume in 1-s, FVC = forced vital capacity forced.

In the MR analysis of the causal relation between birth weight and gut microbiota, we evaluated 207 gut microbiota abundance and 205 gut bacterial pathway abundance. An elevated genetic prediction of birth weight were associated with lower p_Bacteroidetes, c_Bacteroidia, o_Bacteroidales, o_Pasteurellales, f_Pasteurellaceae, g_Haemophilus, s_Haemophilus_parainfluenzae, and s_Bacteroides_plebeius, while higher g_Blautia, g_Subdoligranulum, g_Bilophila, s_Subdoligranulum_unclassified, s_Bilophila_unclassified, s_Ruminococcus_obeum, and s_Coprococcus_catus (Fig. 3, Table S3, Supplemental Digital Content, https://links.lww.com/MD/Q813).

For gut bacterial pathways, our analysis indicated that higher birth weight was causally associated with several key metabolic processes. Specifically, pathways involved in energy metabolism, nucleotide biosynthesis, amino acid metabolism, and certain sugar derivatives showed positive associations with birth weight. In contrast, a pathway related to the biosynthesis of 1,4-dihydroxy-6-naphthoate was negatively associated. These results suggest that birth weight may influence the functional composition of the gut microbiome, particularly pathways related to core metabolism and nucleotide production (Fig. S5, Table S3, Supplemental Digital Content, https://links.lww.com/MD/Q813).

In addition, reverse MR suggested that there was evidence for a causal effect of PRPP.PWY..superpathway.of.histidine..purine..and.pyrimidine.biosynthesis on birth weight (Table S4, Supplemental Digital Content, https://links.lww.com/MD/Q813). Due to insufficient SNPs, we adjusted the threshold to *P* < 1 × 10^−5^. The presence of heterogeneity and pleiotropic effect are shown in Table S6–8 and Figure S2–4, Supplemental Digital Content, https://links.lww.com/MD/Q813.

### 3.3. Effect of gut microbiota on lung function

**Figure 4.**
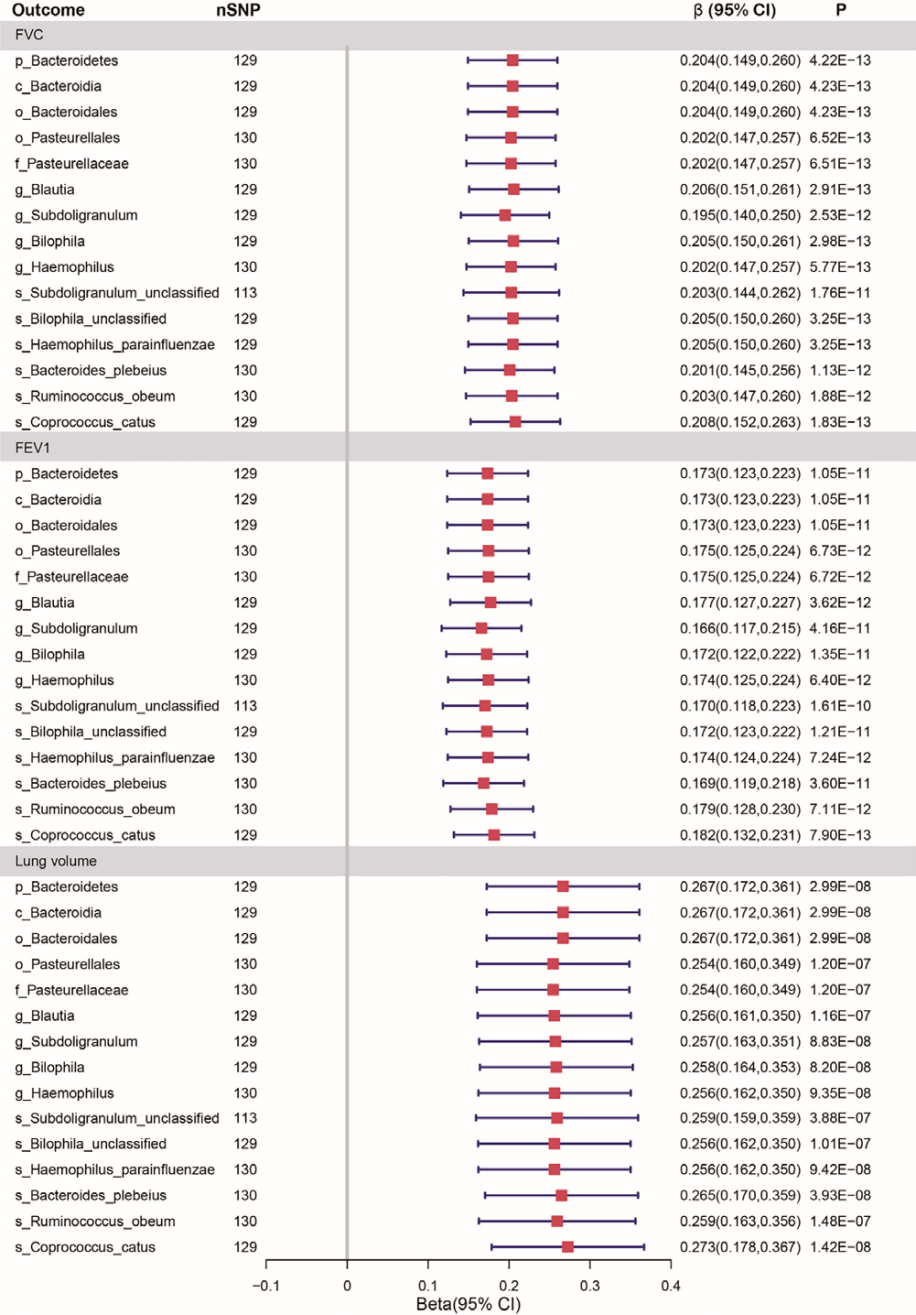
Multivariable Mendelian randomization estimates of the causal association of gut microbiota abundance with lung function after adjusting for birth weight. FEV1 = forced expiratory volume in 1-s, FVC = forced vital capacity forced.

Our forward MR analyses revealed that IVs associated with elevated p_Bacteroidetes, c_Bacteroidia, o_Bacteroidales, o_Pasteurellales, f_Pasteurellaceae, g_Blautia, g_Subdoligranulum, g_Bilophila g_Haemophilus, s_Subdoligranulum_unclassified, s_Bilophila_unclassified, s_Haemophilus_parainfluenzae, s_Bacteroides_plebeius, s_Ruminococcus_obeum, and s_Coprococcus_catus were responsible for increased FVC (Fig. 4, Table S5, Supplemental Digital Content, https://links.lww.com/MD/Q813). Fifteen gut microbiata abundance found to be significantly associated with FEV1 and lung volume (Fig. 4, Table S5, Supplemental Digital Content, https://links.lww.com/MD/Q813).

Meanwhile, 8 pathways were also linked to FVC, FEV1 and lung volume, supporting the implication of microbial metabolism on lung function pathogenesis (Fig. S6, Supplemental Digital Content, https://links.lww.com/MD/Q813).

### 3.4. Mediation MR analysis

We conducted a mediation model to explore the mediating effects of gut microbiota on the relationship between birth weight and lung function. G_Blautia, s_Subdoligranulum_unclassified, s_Ruminococcus_obeum, P441.PWY (superpathway of N-acetylneuraminate degradation) and PWY.7237 (myo-, chiro-, and scillo-inositol degradation) were selected as mediators. These mediators accounted for roughly 17.34 ∼ 28.91% of the effect of LBW on FVC, FEV1, and lung volume (Tables 2–4).

**Table 2:** Proportion of the effect of birth weight on FVC mediated by gut microbiota.

Exposure	β1(SE)	Mediator	β2(SE)	Outcome	β0(SE)	Mediation proportion
Birth weight	0.276 (0.081)	s_Ruminococcus_obeum	0.203 (0.029)	FVC	0.202 (0.026)	27.85% (8.65%, 47.05%)
	0.201 (0.084)	s_Subdoligranulum_unclassified	0.203 (0.03)			20.16% (1.89%, 38.42%)
	0.194 (0.075)	g_Blautia	0.206 (0.028)			19.84% (3.08%, 36.59%)
	0.184 (0.072)	PWY.7237..myo...chiro..and.scillo.inositol.degradation	0.203 (0.029)			18.53% (2.65%, 34.41%)
	0.171 (0.074)	P441.PWY..superpathway.of.N.acetylneuraminate.degradation	0.209 (0.028)			17.71% (1.36%, 34.06%)

FVC = forced vital capacity forced.

**Table 3:** Proportion of the effect of birth weight on FEV1 mediated by gut microbiota.

Exposure	β1(SE)	Mediator	β2(SE)	Outcome	β0(SE)	Mediation proportion
Birth weight	0.276 (0.081)	s_Ruminococcus_obeum	0.179 (0.026)	FEV1	0.171 (0.024)	28.91% (8.69%, 49.13%)
	0.194 (0.075)	g_Blautia	0.177 (0.025)			20.17% (2.93%, 37.41%)
	0.201 (0.084)	s_Subdoligranulum_unclassified	0.170 (0.027)			20.02% (1.67%, 38.37%)
	0.184 (0.072)	PWY.7237..myo...chiro..and.scillo.inositol.degradation	0.174 (0.026)			18.76% (2.48%, 35.04%)
	0.171 (0.074)	P441.PWY..superpathway.of.N.acetylneuraminate.degradation	0.174 (0.025)			17.42% (1.12%, 33.71%)

FEV1 = forced expiratory volume in 1-s.

**Table 4:** Proportion of the effect of birth weight on each lung volume mediated by gut microbiota.

Exposure	β1(SE)	Mediator	β2(SE)	Outcome	β0(SE)	Mediation proportion
Birth weight	0.276 (0.081)	s_Ruminococcus_obeum	0.259 (0.049)	Lung volume	0.267 (0.046)	26.89% (6.32%, 47.46%)
	0.201 (0.084)	s_Subdoligranulum_unclassified	0.259 (0.051)			19.52% (0.66%, 38.37%)
	0.194 (0.075)	g_Blautia	0.256 (0.048)			18.66% (1.7%, 35.62%)
	0.184 (0.072)	PWY.7237..myo...chiro..and.scillo.inositol.degradation	0.265 (0.049)			18.26% (1.54%, 34.97%)
	0.171 (0.074)	P441.PWY..superpathway.of.N.acetylneuraminate.degradation	0.270 (0.048)			17.34% (0.42%, 34.26%)

## 4. Discussion

In this study, we employed 2-sample MR and mediation MR approaches to investigate the potential interactions among birth weight, the gut microbiota and pulmonary function. The results of our study indicated that infants with LBW demonstrated a reduction in FVC, FEV1 and lung volume, independent of confounding factors (measured and unmeasured). Furthermore, our mediation analysis suggested that the impact of LBW on pulmonary function was partially mediated by specific gut microbiota, which revealed the regulation of the gut–lung axis as a potential pathogenic pathway.

Birth weight and pulmonary function are influenced by multiple factors, including genetic and life course events. LBW is regarded as an indicator of intrauterine growth restriction. The DOHaD hypothesis indicates that adverse environmental exposures during early life can lead to permanent physiological, biochemical and epigenetic changes, subsequently increasing the risk of noncommunicable diseases in adulthood, including coronary heart disease, diabetes and impaired pulmonary function.^[24–26]^ Maternal exposures, including smoking, environmental pollutants (such as particulate matter, ozone and carbon monoxide), a pre-pregnancy body mass index < 18.5, inadequate gestational weight gain, pregnancy-induced hypertension, anemia and hypoproteinemia have been demonstrated to alter the intrauterine environment, impairing fetal pulmonary function.^[27]^ The alterations include reduced surfactant production and the number of type-II pneumocytes, decreased alveolar surface area, thickened alveolar blood-gas barriers, and a reduction in lung weight, DNA and protein content.^[28,29]^

Furthermore, animal studies indicated that LBW disrupts pulmonary vascular development, elastic fiber formation and angiogenesis in a time-dependent manner, consequently increasing susceptibility to chronic lung diseases.^[30]^

In accordance with previous studies, our results demonstrated a positive association between LBW with FVC, FEV1 and lung volume, but not FEV1/FVC.^[7,8,31]^ However, some studies have reported a nonlinear relationship between birth weight and lung function.^[32,33]^ Neil J. Saad et al discovered a robust correlation between birth weight and FVC, while the association with FEV1/FVC was weaker. This study indicated that LBW is more closely associated with restrictive lung disease than with airway obstruction.^[9]^ A longitudinal study revealed that children born with LBW were 3 times more likely to develop restrictive patterns of ventilatory impairment in adulthood, in comparison to those with normal birth weight.^[34]^ A higher FVC has been linked to a reduced risk of mortality and a lower prevalence of respiratory symptoms, such as dyspnea.^[35]^ Impaired FEV1 has been linked to several adverse outcomes, including pulmonary disease, cardiovascular disease, and reduced life expectancy.^[36]^ The decline of respiratory muscle strength has a pronounced impact on FVC and FEV1 than on FEV1/FVC, given that the latter also depends on airway patency. Restrictive lung function impairment is associated with an increased prevalence of respiratory symptoms, adverse cardiovascular outcomes, functional limitations, and higher mortality rates.^[37]^

The development of the infant gut microbiota is influenced by multiple factors, including antibiotic exposure, mode of delivery, gestational age and diet.^[38,39]^ The relationship between birth weight and the gut microbiota has been previously reported, with very LBW preterm infants exhibiting reduced microbial diversity at birth.^[40]^ F_pasteurellaceae had a higher relative abundance in LBW which is consistent with our research.^[41]^ The gut microbiota of the LBW group had a greater abundance of g_Haemophilus.^[42]^ An experimental study conducted on pigs revealed that g_Blautia, g_Bifidobacterium, g_Subdoligranulum, and g_Coprococcus 3 were positively associated with lipid metabolic dysfunction and pro-inflammatory response in LBW pigs.^[43]^

**Figure 5.**
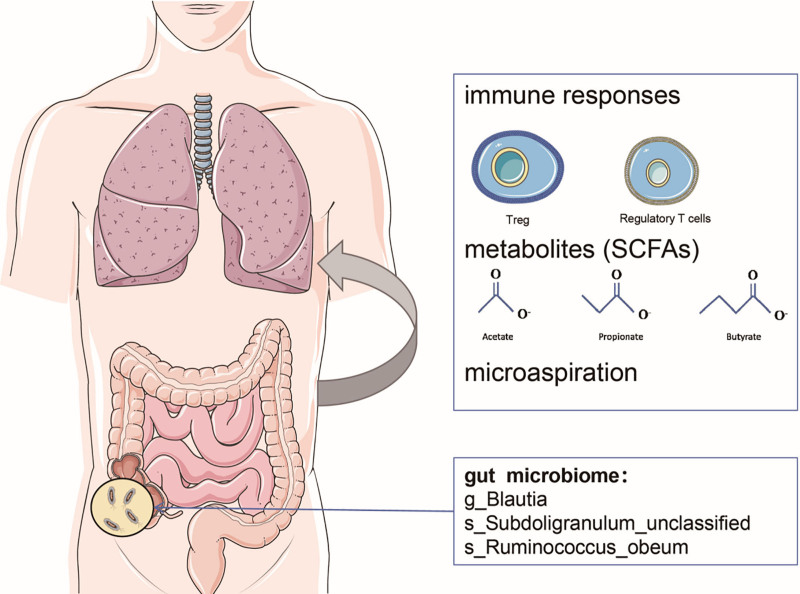
The gut–lung axis and its potential mechanisms. The gut–lung axis may act through 3 major pathways: immune regulation (e.g., modulation of Treg cells and inflammatory signaling), metabolic regulation via SCFAs (acetate, propionate, butyrate), and microbial transfer to the lungs through microaspiration. SCFAs = short-chain fatty acids.

A growing body of evidence suggests that gut microbiota dysbiosis is related to a range of pulmonary dysfunctions, including allergy, asthma, chronic obstructive pulmonary disease, cystic fibrosis, and lung cancer.^[44]^ The gut–lung axis indicates that modulation of the gut microbiota may represent a potential therapeutic avenue for ameliorating pulmonary diseases.^[45,46]^ As a result, maintaining a healthy gut microbiota is crucial for preserving lung health, particularly in older adults.^[47]^ Several pathways have been identified that regulate the gut–lung axis, including immune regulation, metabolic regulation and indirect effects on the respiratory microbiota. (Fig. 5) First, the gut microbiota influences the development of lung disease through immune responses.^[17]^ Regulatory T cells play a central role in maintaining gut homeostasis by preventing inappropriate immune reactions. Certain gut microbiota promote the production of Treg cells, which secrete IL-10, a cytokine essential for limiting lung inflammation.^[48]^ Experimental studies in rats have shown that lipopolysaccharides (LPS) on the surface of gram-negative bacteria can activate toll-like receptor 4, triggering systemic inflammation and affecting lung development.^[49]^ In addition, the gut microbiota can activate immune cells through interactions between toll-like receptor 9 and unmethylated CpG dinucleotides.^[50]^ Second, the gut microbiota regulates the immune system by producing short-chain fatty acids through the fermentation of dietary fiber.^[50]^ Third, microbes from the gastrointestinal tract can reach the lungs through microaspiration. Research by J. C. Madan et al demonstrated that many bacterial species detected in the respiratory tract first appeared in the intestine, and fluctuations in bacterial abundance occurred similarly at both sites.^[51]^

We identified positive associations between p_Bacteroidetes, c_Bacteroidia, o_Bacteroidales, and s_Bacteroides_plebeius with FVC, FEV1, and lung volume. The metabolic activities of the aforementioned gut microbiota contribute to maintaining intestinal ecological balance by producing short-chain fatty acids as metabolites during the fermentation of dietary fiber and resistant starch.^[52]^ These metabolites can directly affect lung health through the bloodstream, facilitated by free fatty acid receptors or epigenetic regulation of immune cells.^[53,54]^ A recent study has also reported decreased abundance of p_Bacteroidetes in the lung during the progression of idiopathic pulmonary fibrosis, which aligns with changes in gut microbiota.^[55]^ However, 1 study presented contradictory findings, proposing that pulmonary dysbiosis, characterized by elevated levels of p_Bacteroidetes, was associated with reduced FVC.^[56]^

Our results indicated that s_Coprococcus_catus, g_Blautia, and s_Ruminococcus_obeum in the family Lachnospiraceae are positively associated with lung function. Notably, g_Blautia and s_Ruminococcus_obeum mediated the effects of LBW on lung function. s_Coprococcus_catus can metabolize various carbohydrate substrates, such as β-glucans, to produce butyrate, formate and lactate. Butyrate, as a SCFA, plays a crucial role in maintaining gut health, providing energy, and modulating immune responses.^[57]^ Research by Sijia Li et al revealed that the abundance of s_Coprococcus_catus were decreased in COVID-19 patients compared with healthy controls.^[58]^ It has been demonstrated that g_Blautia can utilize carbon dioxide to produce acetate, which serves as a secondary energy source for intestinal epithelial cells and modulates immune system functions.^[59]^ A negative correlation between g_Blautia abundance and visceral fat has been reported in a previous study.^[60]^ Visceral fat is considered to affect lung function through endocrine pathways, diaphragm movement, and inflammation induction.^[61,62]^ COVID-19 patients have exhibited reduced g_Blautia levels in their gut microbiota compared with healthy controls.^[63]^ s_Ruminococcus_obeum has been reclassified as s_Blautia_obeum.^[64]^ S_Blautia_obeum has been demonstrated to possess the potential to inhibit the proliferation of Clostridium perfringens, thus preventing the colonization of intestinal pathogens.^[65]^ S_Blautia_obeum has been associated with diseases, including gestational diabetes,^[66]^ depression,^[67]^ and primary sclerosing cholangitis.^[68]^ S_Blautia_obeum can utilize mucin-derived sugars, such as fucose, to produce propionate.^[69]^ Propionate serves as a crucial energy source and immunomodulator, playing a pivotal role in the preservation of intestinal health.^[69,70]^ However, it can also induce epithelial-mesenchymal transition, potentially contributing to fibrotic diseases.^[71]^ Furthermore, a study revealed an increase of s_Blautia_obeum in the control group compared to tuberculosis patients.^[72]^

Within the family Ruminococcaceae, g_Subdoligranulum and s_Subdoligranulum_unclassified are positively correlated with lung function. In particular, s_Subdoligranulum_unclassified mediates the effects of LBW on lung function. A prospective study investigating whether gut dysbiosis influences long-term COVID-19 outcomes revealed a decreasing trend in the relative abundance of Subdoligranulum across healthy controls, asymptomatic individuals, and symptomatic individuals.^[73]^ In pulmonary and critical care medicine inpatients, gut dysbiosis was observed, with g_Blautia, g_Subdoligranulum, g_Enterococcus, and g_Klebsiella distinguishing hospitalized patients from healthy controls.^[74]^ S_Subdoligranulum_unclassified is a beneficial bacterium capable of producing butyrate and is closely associated with T cell immune activation.^[75]^

Currently, no reported associations between o_Pasteurellales or f_Pasteurellaceae and lung function were reported. The abundance of coal workers’ pneumoconiosis patients shows an increased presence of pro-inflammatory microorganisms, such as g_Haemophilus.^[76]^ The relative abundance of Haemophilus is higher in the neonatal respiratory distress syndrome group compared to the preterm infants group.^[77]^ These findings are at odds with our study, which indicated a positive correlation between g_Haemophilus and FVC, FEV1, and lung volume. Previous studies suggest that the effect of s_Haemophilus_parainfluenzae impact on lung function remains controversial. S_Haemophilus_parainfluenzae is enriched in healthy control groups compared to tuberculosis groups.^[72]^ Additionally, s_Haemophilus_parainfluenzae is significantly enriched in the gut samples of patients with lung adenocarcinoma.^[78]^

G_Bilophila is an anaerobic, gram-negative, bile-resistant, catalase-positive rod that exists as part of the normal gut microbiota in human feces. The abundance of g_Bilophila was found to increase in COVID-19 patients, potentially indicating that gut microbiota dysbiosis plays a role in the progression of COVID-19.^[79]^ Our conclusion contradicts this finding and should be interpreted with caution. There are no related reports on s_Bilophila_unclassified.

Our study also indicates that LBW contributes to increased FVC, FEV1, and lung volume through the pathways “P441.PWY: Superpathway of N-acetylneuraminate degradation” and “PWY.7237: myo-, chiro-, and scyllo-inositol degradation,” respectively. However, the mechanisms underlying the interactions of these bacterial pathways remain unknown.

The gut–lung axis is a complex and critical biological system, where the gut microbiota influences lung health and disease through various mechanisms. Strategies such as dietary modulation, probiotic supplementation, direct supplementation of SCFA, antibiotic treatment, and fecal microbiota transplantation can improve the adverse effects of LBW on lung disease prognosis by regulating the gut microbiota. Future research should further explore the specific mechanisms of the gut–lung axis in restrictive lung diseases and potential therapeutic strategies.

Our study has notable strengths. First, it is the first 2-step MR analysis using recently published GWAS data to evaluate the causal mediating effect of gut microbiota on the relationship between birth weight and lung function (FVC, FEV1, FEV1/FVC and lung capacity). Second, we employed 5 complementary analyses to adjust for pleiotropic bias and to confirm the robustness of our findings. Thirdly, the use of large-scale GWAS data enhances the accuracy of causal relationships between risk factor exposures and disease outcomes compared to individual-level observational studies.

However, this study also has limitations. First, the GWAS data were collected from individuals of European ancestry, which might restrict the applicability of these results to other ethnic groups. Second, genetic variations primarily reflect lifelong exposure rather than short-term intervention, so further clinical validation is required to determine the potential utility of gut microbiota as biomarkers. Third, we used 2-sample MR analysis to assess the linear effect association between birth weight and lung function, lacking exploration of nonlinear associations. Fourth, assumption 3 of the IVs is not ascertained, and horizontal pleiotropy may introduce some bias. Although our sensitivity analyses help to reduce this concern, they cannot completely exclude it. Fifth, lung volume was derived from CT imaging in the UK Biobank rather than measured by plethysmography, which remains the gold standard for assessing total lung capacity. While CT-based lung volumes have been shown to correlate strongly with plethysmographic total lung capacity in validation studies, they may not fully capture restrictive physiology in the same way. Sixth, neonatal gut microbiota datasets are currently unavailable, and we therefore relied on adult gut microbiota GWAS data as proxies. While adult microbiota are influenced by diet, environment, and medication use, making the causal pathway from birth weight to later microbial profiles speculative, our findings provide exploratory evidence that highlights potential mechanisms. Lastly, the mediation proportions carry wide confidence intervals, and thus the observed effects should be interpreted as indicative rather than definitive.

## 5. Conclusion

In this study, a 2-step MR design was used to explore the causal mediating effect of gut microbiota on the relationship between birth weight and lung function. These findings suggested that prevention and intervention targeting gut microbiota dysbiosis could improve lung function impairment in individuals with LBW.

## Acknowledgments

We are grateful for the suggestions of the editor and anonymous reviewers. We also thank for the open data set OpenGWAS.

## Author contributions

**Conceptualization:** Limin Cao.

**Data curation:** Limin Cao, Yun Li, Qiwei Wang, Keyi Fan.

**Formal analysis:** Limin Cao, Yun Li, Keyi Fan, Zhenglong Li.

**Investigation:** Limin Cao, Keyi Fan, Yahui Wen.

**Methodology:** Limin Cao, Yaochen Zhang, Keyi Fan, Xinyue Zhang.

**Project administration:** Limin Cao, Yaochen Zhang, Xinhua Zhang.

**Resources:** Limin Cao, Yaochen Zhang, Xinhua Zhang.

**Supervision:** Xinhua Zhang.

**Validation:** Xinhua Zhang.

**Visualization:** Limin Cao.

**Writing – original draft:** Limin Cao.

**Writing – review & editing:** Qiwei Wang, Xinhua Zhang.

## Supplementary Material


