# Supplementary material for: Association of birth weight with lung function and the mediating role of gut microbiota: A STROBE 2-step Mendelian randomization study
Source: Medicine (Baltimore). 2025 Dec 26;104(52):e46294. doi: 10.1097/MD.0000000000046294 (PMC12747014; doi:10.1097/MD.0000000000046294)
Supplement: Supplementary file 1 [file medi-104-e46294-s001.pdf]

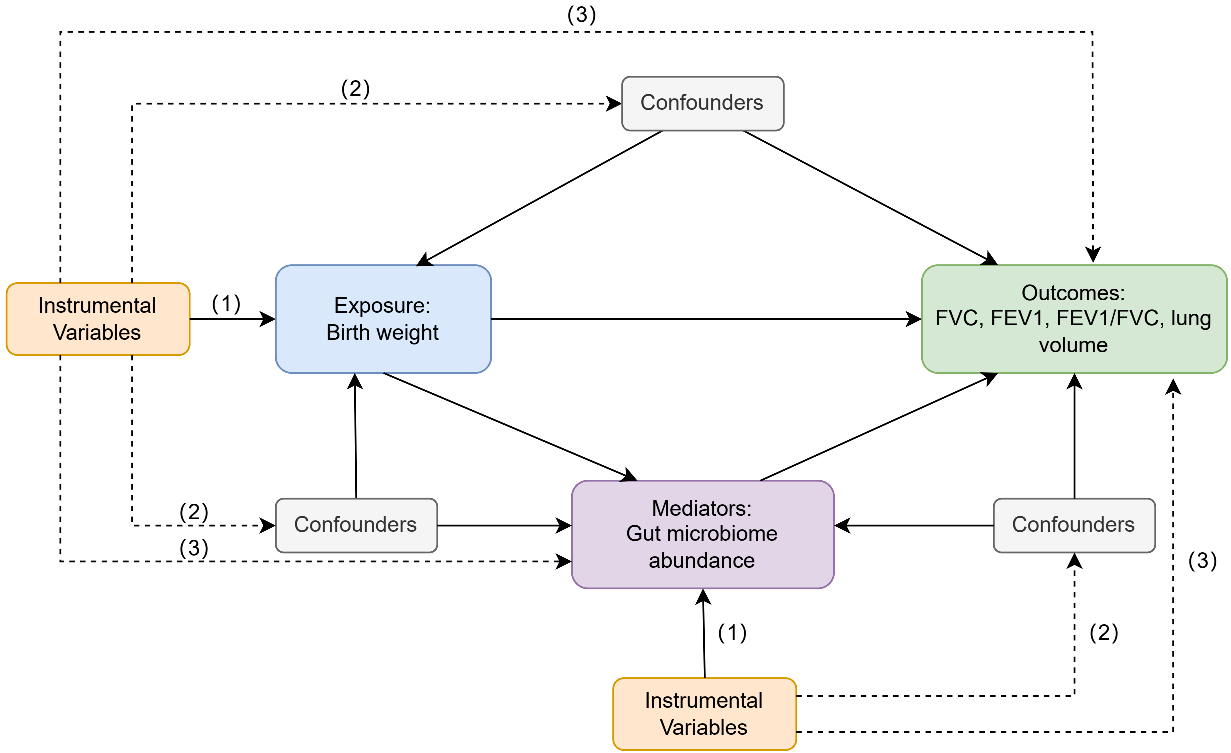
(3)
(2)
Confounders
Instrumental Variables
(1)
Exposure: Birth weight
Outcomes: FVC, FEV1, FEV1/FVC, lung volume
(2)
Confounders
(3)
Mediators: Gut microbiome abundance
Confounders
(3)
(1)
(2)
Instrumental Variables

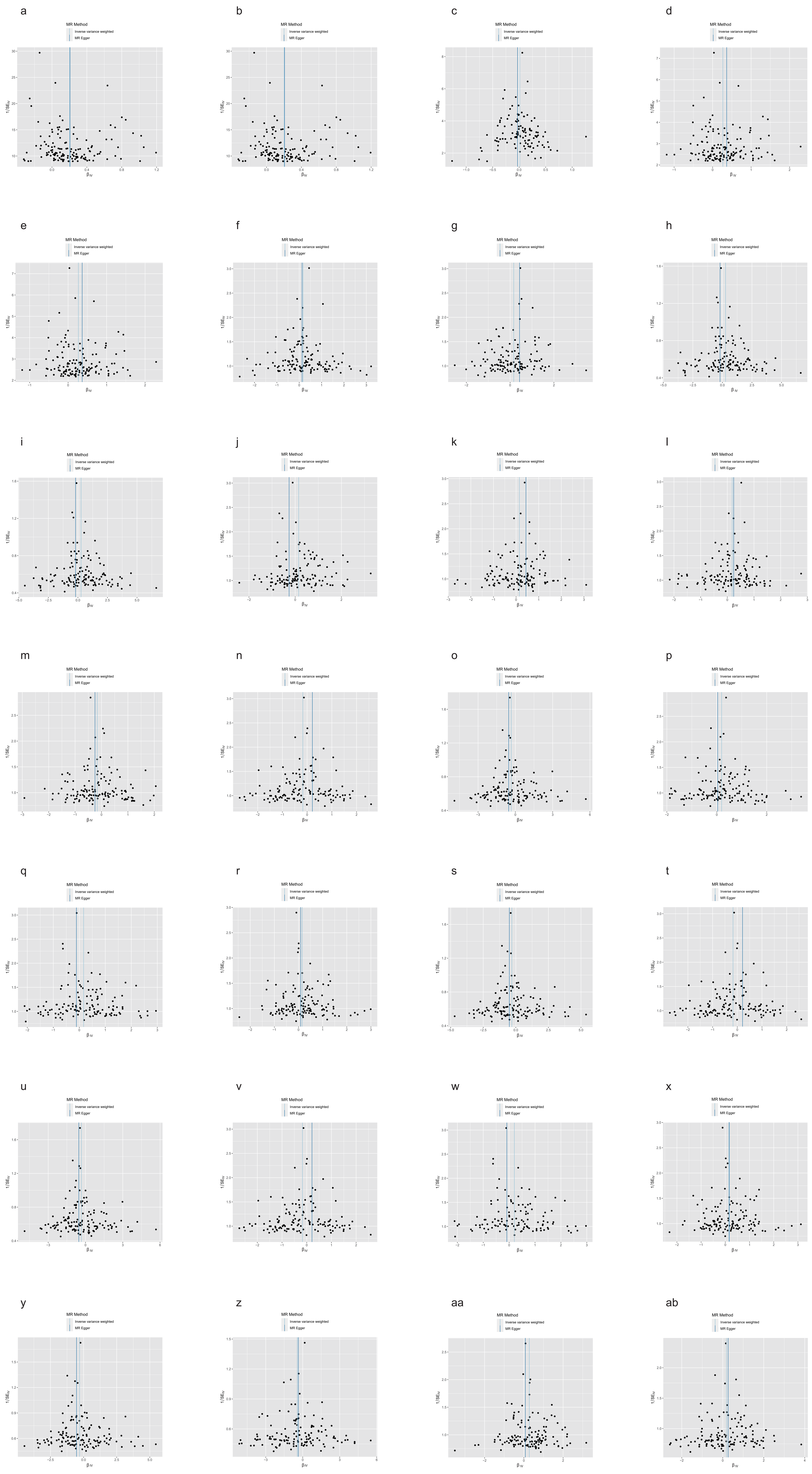
a
b
c
d
e
f
g
h
i
j
k
l
m
n
o
p
q
r
s
t
u
v
w
x
y
z
aa
ab
MR Method
Inverse variance weighted
MR Egger
1/SE_IV
β_IV

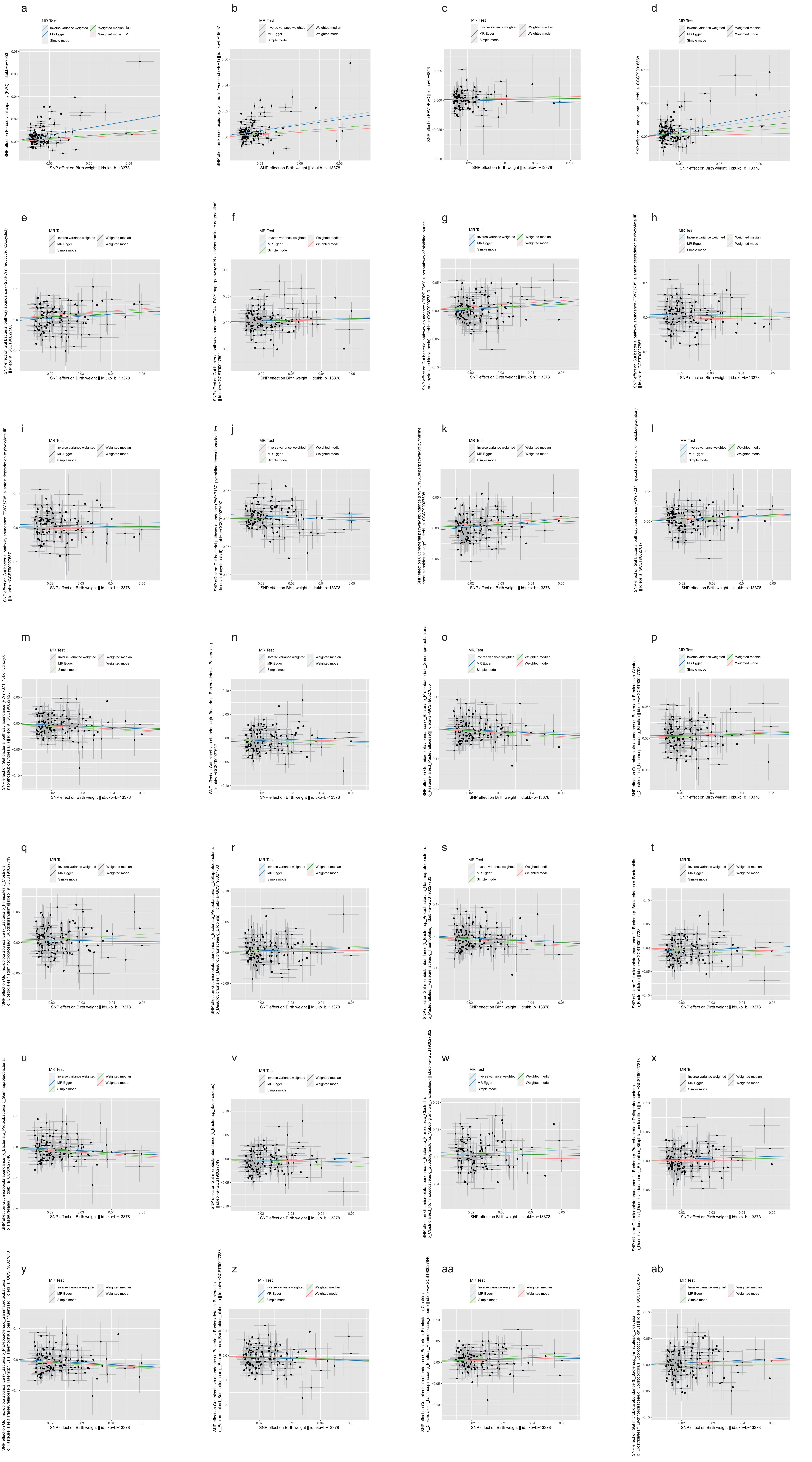
a
b
c
d
e
f
g
h
i
j
k
l
m
n
o
p
q
r
s
t
u
v
w
x
y
z
aa
ab
MR Test
Inverse variance weighted
MR Egger
Simple mode
Weighted median
Weighted mode
SNP effect on Birth weight || id:ukb-b-13378

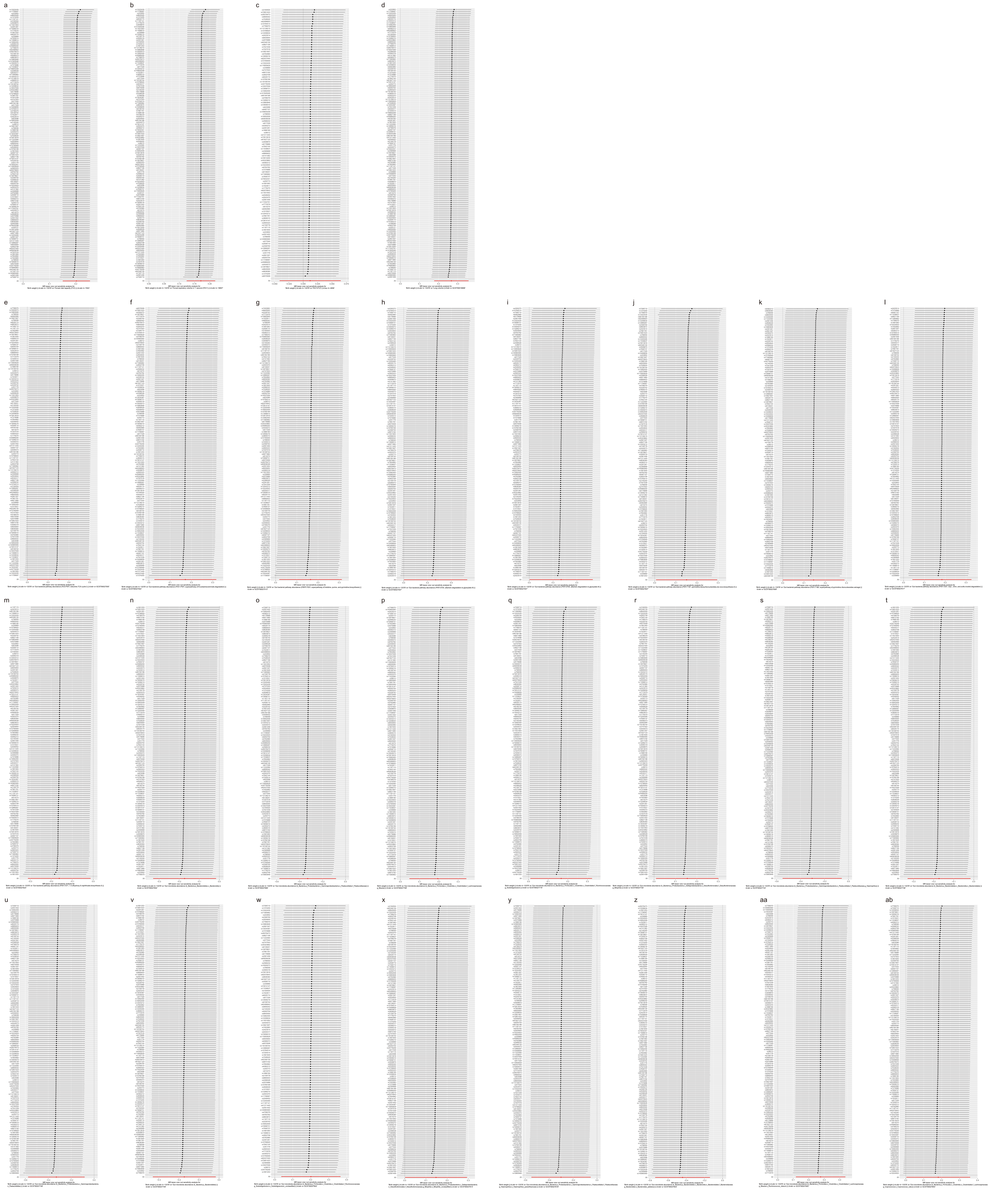
a
b
c
d
e
f
g
h
i
j
k
l
m
n
o
p
q
r
s
t
u
v
w
x
y
z
aa
ab

Supplemental text 3：STROBE-MR checklist of recommended items to address in reports of Mendelian randomization studies

Item No.	Section		Checklist item	Page No.	Relevant text from manuscript
1	**TITLE and ABSTRACT**		Indicate Mendelian randomization (MR) as the study's design in the title and/or the abstract if that is a main purpose of the study	2	The term "Mendelian randomization" was incorporated into the abstract.
	**INTRODUCTION**				
2	**Background**		Explain the scientific background and rationale for the reported study. What is the exposure? Is a potential causal relationship between exposure and outcome plausible? Justify why MR is a helpful method to address the study question	3,4	Introduction, Line 50-84.
3	**Objectives**		State specific objectives clearly, including pre-specified causal hypotheses (if any). State that MR is a method that, under specific assumptions, intends to estimate causal effects	5	Introduction, Line 86-89.
	**METHODS**				
4	**Study design and data sources**		Present key elements of the study design early in the article. Consider including a table listing sources of data for all phases of the study. For each data source contributing to the analysis, describe the following:		
		a)	Setting: Describe the study design and the underlying population, if possible. Describe the setting, locations, and relevant dates, including periods of recruitment, exposure, follow-up, and data collection, when available.	5-6,	Methods, Line 91-112;<br>Figure 1.
		b)	Participants: Give the eligibility criteria, and the sources and methods of selection of participants. Report the sample size, and whether any power or sample size calculations were carried out prior to the main analysis	6,7	Methods, Line 106-114;<br>Table1;

		c)	Describe measurement, quality control and selection of genetic variants	6	Methods, Line 115-121.
		d)	For each exposure, outcome, and other relevant variables, describe methods of assessment and diagnostic criteria for diseases	5,6	Methods, Line 105-114.
		e)	Provide details of ethics committee approval and participant informed consent, if relevant		
5	**Assumptions**		Explicitly state the three core IV assumptions for the main analysis (relevance, independence and exclusion restriction) as well assumptions for any additional or sensitivity analysis	5,6	Methods, Line 107-114.
6	**Statistical methods: main analysis**		Describe statistical methods and statistics used		
		a)	Describe how quantitative variables were handled in the analyses (i.e., scale, units, model)	N/A	In our MR analysis, birth weight, FVC, FEV1, FEV1/FVC had a unit of 1-SD and was standardized in its GWAS.
		b)	Describe how genetic variants were handled in the analyses and, if applicable, how their weights were selected	5,6	Methods, Line 106-121.
		c)	Describe the MR estimator (e.g. two-stage least squares, Wald ratio) and related statistics. Detail the included covariates and, in case of two-sample MR, whether the same covariate set was used for adjustment in the two samples	6	Methods, Line 115-121.
		d)	Explain how missing data were addressed		N/A
		e)	If applicable, indicate how multiple testing was addressed		
7	**Assessment of assumptions**		Describe any methods or prior knowledge used to assess the assumptions or justify their validity	5,6	Methods, Line 86-121
8	**Sensitivity analyses and additional analyses**		Describe any sensitivity analyses or additional analyses performed (e.g. comparison of effect estimates from different approaches, independent	6	Methods, Line 117-121.

			replication, bias analytic techniques, validation of instruments, simulations)		
9	**Software and pre-registration**				
		a)	Name statistical software and package(s), including version and settings used	6	Methods, Line 120-121.
		b)	State whether the study protocol and details were pre-registered (as well as when and where)	N/A	
	**RESULTS**				
10	**Descriptive data**				
		a)	Report the numbers of individuals at each stage of included studies and reasons for exclusion. Consider use of a flow diagram	N/A	
		b)	Report summary statistics for phenotypic exposure(s), outcome(s), and other relevant variables (e.g. means, SDs, proportions)	N/A	
		c)	If the data sources include meta-analyses of previous studies, provide the assessments of heterogeneity across these studies	N/A	
		d)	For two-sample MR:<br>i. Provide justification of the similarity of the genetic variant-exposure associations between the exposure and outcome samples<br>ii. Provide information on the number of individuals who overlap between the exposure and outcome studies	N/A	There was minimal ethic heterogeneity because all the samples used here were of European ancestry. However, the study did not report any information regarding overlapping populations between the exposure and outcome in our study.
11	**Main results**				
		a)	Report the associations between genetic variant and exposure, and between genetic variant and outcome, preferably on an interpretable scale	6-17	

		b)	Report MR estimates of the relationship between exposure and outcome, and the measures of uncertainty from the MR analysis, on an interpretable scale, such as odds ratio or relative risk per SD difference	6-17	
		c)	If relevant, consider translating estimates of relative risk into absolute risk for a meaningful time period	N/A	
		d)	Consider plots to visualize results (e.g. forest plot, scatterplot of associations between genetic variants and outcome versus between genetic variants and exposure)		Figures 2-4 and Figure S2-S4.
12	**Assessment of assumptions**				
		a)	Report the assessment of the validity of the assumptions		Table S6-8
		b)	Report any additional statistics (e.g., assessments of heterogeneity across genetic variants, such as I^2, Q statistic or E-value)		Table S6-8
13	**Sensitivity analyses and additional analyses**				
		a)	Report any sensitivity analyses to assess the robustness of the main results to violations of the assumptions		Table S2-5.
		b)	Report results from other sensitivity analyses or additional analyses		Table S2-5.
		c)	Report any assessment of direction of causal relationship (e.g., bidirectional MR)	7	Line 142-145.
		d)	When relevant, report and compare with estimates from non-MR analyses	9-15	Discussion section.
		e)	Consider additional plots to visualize results (e.g., leave-one-out analyses)		Figure S4
	**DISCUSSION**				

14	**Key results**		Summarize key results with reference to study objectives	9	Discussion, Line 147-180.
15	**Limitations**		Discuss limitations of the study, taking into account the validity of the IV assumptions, other sources of potential bias, and imprecision. Discuss both direction and magnitude of any potential bias and any efforts to address them	15	Discussion, Line 317-323.
16	**Interpretation**				
		a)	Meaning: Give a cautious overall interpretation of results in the context of their limitations and in comparison with other studies	9-15	Discussion, Line 174-323.
		b)	Mechanism: Discuss underlying biological mechanisms that could drive a potential causal relationship between the investigated exposure and the outcome, and whether the gene-environment equivalence assumption is reasonable. Use causal language carefully, clarifying that IV estimates may provide causal effects only under certain assumptions	9-15	Discussion, Line 174-323.
		c)	Clinical relevance: Discuss whether the results have clinical or public policy relevance, and to what extent they inform effect sizes of possible interventions	9-15	Discussion, Line 174-323.
17	**Generalizability**		Discuss the generalizability of the study results (a) to other populations, (b) across other exposure periods/timings, and (c) across other levels of exposure	15	Discussion, Line 317-319.
	**OTHER INFORMATION**				
18	**Funding**		Describe sources of funding and the role of funders in the present study and, if applicable, sources of funding for the databases and original study or studies on which the present study is based	17	Funding, Line 339-341.
19	**Data and data sharing**		Provide the data used to perform all analyses or report where and how the data can be accessed, and reference these sources in the article. Provide the statistical code needed to reproduce the results in the article, or report whether the code is publicly	17	Availability of data and materials, Line 342-345.

		accessible and if so, where		
20	**Conflicts of Interest**	All authors should declare all potential conflicts of interest	18	Competing Interests, Line 352-353 .

This checklist is copyrighted by the Equator Network under the Creative Commons Attribution 3.0 Unported (CC BY 3.0) license.

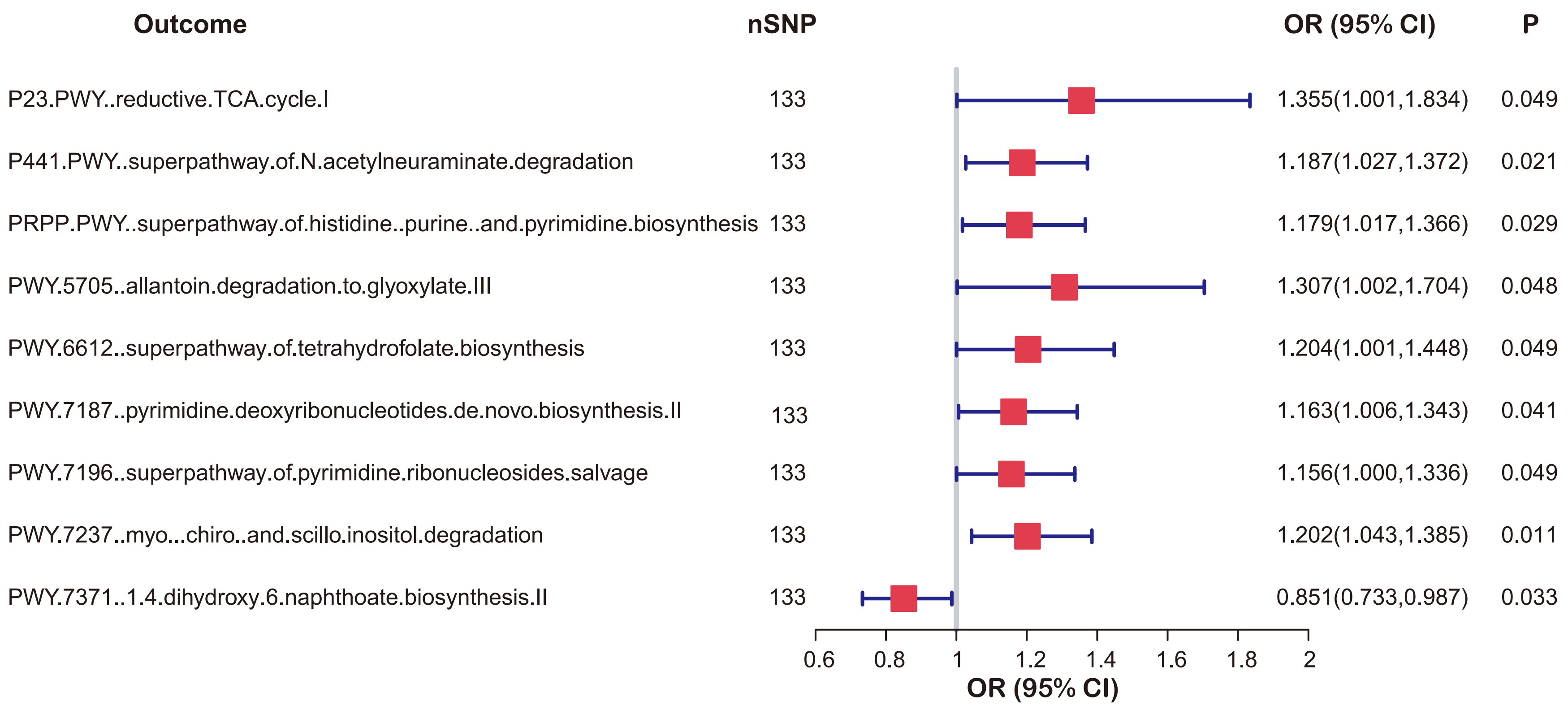
Outcome
nSNP
OR (95% CI)
P
P23.PWY..reductive.TCA.cycle.I
133
1.355(1.001,1.834)
0.049
P441.PWY..superpathway.of.N.acetylneuraminate.degradation
133
1.187(1.027,1.372)
0.021
PRPP.PWY..superpathway.of.histidine..purine..and.pyrimidine.biosynthesis
133
1.179(1.017,1.366)
0.029
PWY.5705..allantoin.degradation.to.glyoxylate.III
133
1.307(1.002,1.704)
0.048
PWY.6612..superpathway.of.tetrahydrofolate.biosynthesis
133
1.204(1.001,1.448)
0.049
PWY.7187..pyrimidine.deoxyribonucleotides.de.novo.biosynthesis.II
133
1.163(1.006,1.343)
0.041
PWY.7196..superpathway.of.pyrimidine.ribonucleosides.salvage
133
1.156(1.000,1.336)
0.049
PWY.7237..myo...chiro..and.scillo.inositol.degradation
133
1.202(1.043,1.385)
0.011
PWY.7371..1.4.dihydroxy.6.naphthoate.biosynthesis.II
133
0.851(0.733,0.987)
0.033
0.6
0.8
1
1.2
1.4
1.6
1.8
2
OR (95% CI)

Outcome	nSNP	β (95% CI)	P
FVC			
P23.PWY..reductive.TCA.cycle.I	129	0.203(0.148,0.259)	4.99E−13
P441.PWY..superpathway.of.N.acetylneuraminate.degradation	129	0.209(0.154,0.264)	9.40E−14
PWY.5705..allantoin.degradation.to.glyoxylate.III	129	0.207(0.152,0.262)	1.44E−13
PWY.6612..superpathway.of.tetrahydrofolate.biosynthesis	130	0.213(0.159,0.267)	9.80E−15
PWY.7187..pyrimidine.deoxyribonucleotides.de.novo.biosynthesis.II	129	0.201(0.146,0.256)	8.34E−13
PWY.7196..superpathway.of.pyrimidine.ribonucleosides.salvage	130	0.2(0.145,0.254)	9.93E−13
PWY.7237..myo...chiro..and.scillo.inositol.degradation	129	0.203(0.147,0.26)	1.40E−12
PWY.7371..1.4.dihydroxy.6.naphthoate.biosynthesis.II	129	0.2(0.145,0.255)	1.50E−12
FEV1			
P23.PWY..reductive.TCA.cycle.I	129	0.174(0.124,0.224)	8.39E−12
P441.PWY..superpathway.of.N.acetylneuraminate.degradation	129	0.174(0.124,0.224)	9.64E−12
PWY.5705..allantoin.degradation.to.glyoxylate.III	129	0.176(0.126,0.225)	4.59E−12
PWY.6612..superpathway.of.tetrahydrofolate.biosynthesis	130	0.181(0.132,0.23)	3.79E−13
PWY.7187..pyrimidine.deoxyribonucleotides.de.novo.biosynthesis.II	129	0.176(0.126,0.226)	4.10E−12
PWY.7196..superpathway.of.pyrimidine.ribonucleosides.salvage	130	0.172(0.122,0.221)	1.18E−11
PWY.7237..myo...chiro..and.scillo.inositol.degradation	129	0.174(0.123,0.225)	1.93E−11
PWY.7371..1.4.dihydroxy.6.naphthoate.biosynthesis.II	129	0.169(0.119,0.219)	3.44E−11
Lung volume			
P23.PWY..reductive.TCA.cycle.I	129	0.262(0.167,0.356)	5.43E−08
P441.PWY..superpathway.of.N.acetylneuraminate.degradation	129	0.27(0.176,0.364)	1.78E−08
PWY.5705..allantoin.degradation.to.glyoxylate.III	129	0.262(0.168,0.356)	4.88E−08
PWY.6612..superpathway.of.tetrahydrofolate.biosynthesis	130	0.275(0.183,0.368)	5.13E−09
PWY.7187..pyrimidine.deoxyribonucleotides.de.novo.biosynthesis.II	129	0.259(0.165,0.353)	7.01E−08
PWY.7196..superpathway.of.pyrimidine.ribonucleosides.salvage	130	0.261(0.167,0.355)	5.01E−08
PWY.7237..myo...chiro..and.scillo.inositol.degradation	129	0.265(0.168,0.361)	6.83E−08
PWY.7371..1.4.dihydroxy.6.naphthoate.biosynthesis.II	129	0.259(0.164,0.353)	8.77E−08

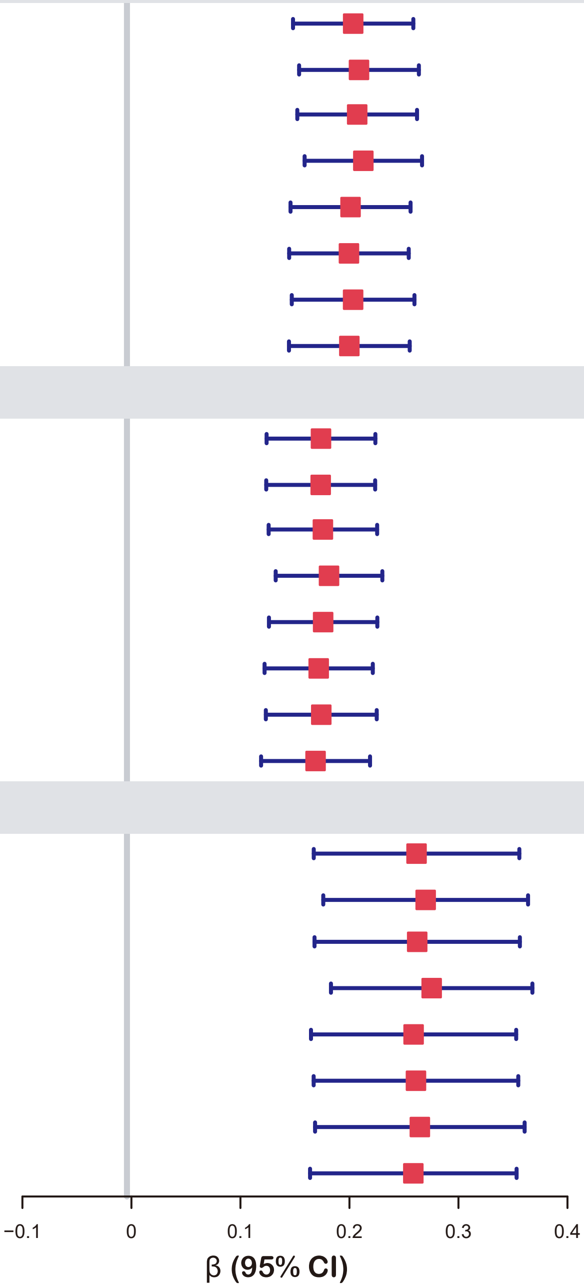

Supplementary table 1: Characteristics of SNPs used as instrumental variables for birth weight

	SNP	chr.exposure	pos.exposure	effect_allele.exposure	other_allele.exposure	beta.exposure	eaf.exposure	se.exposure	pval.exposure	r2	F
1	rs10913200	1	176521655	A	G	-0.0533093	0.028623	0.00807933	4.19952E-11	0.00015803	41.39928132
2	rs823096	1	205679887	T	G	-0.015135	0.448547	0.00272065	2.69998E-08	0.000113321	29.6855956
3	rs7541039	1	214176779	T	C	0.0189341	0.260431	0.00307797	7.69999E-10	0.000138099	36.17728147
4	rs80184186	1	43428581	G	A	-0.0292003	0.096521	0.00457782	1.79999E-10	0.000148711	38.95779159
5	rs35989200	1	161574305	G	A	-0.0177192	0.230717	0.00322898	4.09996E-08	0.000111451	29.19561281
6	rs1776270	1	150369337	A	G	0.0159634	0.631655	0.00280379	0.000000012	0.000118581	31.06363325
7	rs141845046	1	154987704	T	C	0.0879853	0.021842	0.00930975	3.40017E-21	0.000330789	86.67235054
8	rs12043212	1	155964698	G	A	0.0189714	0.637119	0.00280486	1.29987E-11	0.000166423	43.598452
9	rs3806315	1	214724668	A	G	0.0179535	0.593763	0.00275595	7.29962E-11	0.000155497	40.73555473
10	rs3008870	1	67418027	T	C	-0.0196624	0.728887	0.0030374	9.60064E-11	0.000152797	40.02812094
11	rs78444298	1	184672098	A	G	-0.0544097	0.019389	0.00985844	3.40001E-08	0.000112573	29.48960566
12	rs1991083	2	23887437	T	C	0.0232327	0.680137	0.00291195	1.50003E-15	0.00023485	61.52859825
13	rs8179686	2	109240242	T	C	-0.0169469	0.455937	0.00271393	4.30002E-10	0.000142483	37.3260201
14	rs9636364	2	111992435	A	G	0.0168765	0.543484	0.0027066	4.49997E-10	0.000141331	37.02406981
15	rs72790949	2	43858032	C	T	0.017025	0.25245	0.00312085	4.90004E-08	0.000109401	28.65844456
16	rs2140240	2	226988137	A	G	0.0192043	0.275074	0.00318211	1.6E-09	0.000147086	38.53180061
17	rs7606737	2	46625781	G	A	0.017146	0.658885	0.00285945	0.000000002	0.00013215	34.61853704
18	rs1522811	2	226992454	A	C	0.0210036	0.225712	0.00323899	8.9002E-11	0.000154197	40.39493054
19	rs1374205	2	46484188	C	T	0.0445365	0.699414	0.00306513	7.8001E-48	0.000833998	218.6315477
20	rs13034367	2	9673372	A	T	0.026827	0.304702	0.00293527	6.29941E-20	0.000304944	79.89842125
21	rs2070988	3	38437835	G	A	-0.0158848	0.373068	0.00279972	1.40001E-08	0.000118033	30.91992569
22	rs11708067	3	123065778	G	A	0.0401315	0.242935	0.00314716	3.10027E-37	0.000592412	155.262523
2	rs1332	3	15679	G	A	-0.0499	0.4042	0.002	7.7001	0.0011	314.62

3	2435		5468			086	19	76485	6E-73	99732	31941
24	rs7374686	3	67075271	G	A	-0.0288745	0.891157	0.00442974	7.10068E-11	0.000161739	42.37104333
25	rs13082455	3	148618975	T	G	-0.0205242	0.607137	0.00276435	1.10002E-13	0.000200951	52.64569028
26	rs9827156	3	183380900	A	G	-0.0213473	0.262196	0.00307494	3.90032E-12	0.000176312	46.18966272
27	rs2306700	3	142123841	T	C	0.024039	0.135394	0.00395769	1.2E-09	0.000135295	35.44250907
28	rs62346130	4	145578799	T	C	0.0237116	0.435161	0.00272323	3.10027E-18	0.000276393	72.41551985
29	rs1015521	4	106115450	T	G	0.0247758	0.330407	0.00287395	6.59933E-18	0.00027161	71.16209595
30	rs149600208	4	134772442	T	C	0.0921723	0.01677	0.0107151	7.8001E-18	0.000280168	73.40505431
31	rs2061456	4	17998426	A	C	0.0316666	0.260587	0.00307803	8.00018E-25	0.000386432	101.2571727
32	rs12656216	5	36160668	A	G	0.019908	0.785045	0.00330141	1.6E-09	0.00013376	35.04053283
33	rs10037009	5	57051724	T	C	-0.0303903	0.156516	0.00371712	2.90001E-16	0.000243857	63.88909276
34	rs9293754	5	77912801	T	C	0.0163939	0.430307	0.00272781	1.89998E-09	0.000131769	34.51885062
35	rs329121	5	133863352	C	G	0.0182509	0.581371	0.00274007	2.70023E-11	0.000162137	42.47534403
36	rs71585962	5	157844105	A	G	0.0188506	0.362572	0.00281105	1.99986E-11	0.00016425	43.02910692
37	rs72813916	5	158437819	C	T	-0.0240127	0.13742	0.00394679	1.2E-09	0.000136698	35.81013254
38	rs6873192	5	67598184	G	A	-0.0158487	0.515848	0.0027062	4.70002E-09	0.000125464	32.86703303
39	rs2048452	5	90567180	A	C	-0.0231891	0.876809	0.0041142	0.000000017	0.000116167	30.4310865
40	rs2747503	6	15066121	T	C	-0.0179767	0.314835	0.00287591	4.09996E-10	0.000139421	36.52362801
41	rs1187115	6	34172055	A	C	-0.0302476	0.8301	0.0035991	4.30031E-17	0.000258069	67.61342969
42	rs6570508	6	142713842	A	G	-0.0208491	0.303939	0.002939	1.29987E-12	0.000183924	48.18406979
43	rs7772579	6	152042502	C	A	-0.0308134	0.276637	0.00302185	1.99986E-24	0.000379993	99.56944799
44	rs3095318	6	31088145	A	T	0.0166808	0.26669	0.00304653	4.39997E-08	0.000108832	28.50956532
45	rs9382212	6	53330801	C	T	-0.0285053	0.270829	0.00303078	5.19996E-21	0.000320927	84.08730311
46	rs12206634	6	109353407	A	G	0.0370043	0.121396	0.0041464	4.49987E-19	0.0002921	76.53217469
47	rs9399373	6	141878669	A	T	0.0236772	0.745016	0.00313203	4.00037E-14	0.000212995	55.80164036

48	rs6935954	6	26255451	G	A	-0.0155799	0.57798	0.00273512	0.000000012	0.000118415	31.0200036
49	rs12205495	6	35484850	A	T	0.0260056	0.218355	0.00328755	2.60016E-15	0.000230853	60.48141738
50	rs62437400	6	158785893	T	C	0.0196226	0.234728	0.00321984	1.09999E-09	0.000138332	36.23841078
51	rs2934844	6	166142456	T	A	0.0179273	0.670329	0.00290095	6.4E-10	0.000142046	37.2113528
52	rs7766070	6	20686573	A	C	-0.0327367	0.26388	0.00306233	1.10002E-26	0.000416346	109.0990536
53	rs2395668	6	37105893	G	A	-0.0218927	0.16424	0.00364894	0.000000002	0.00013158	34.46922799
54	rs35323862	6	127428232	T	C	0.0199067	0.236387	0.00318465	4.09996E-10	0.000143062	37.47769045
55	rs836559	7	6448763	C	G	0.014909	0.458959	0.00271301	3.89996E-08	0.00011039	28.91773545
56	rs6971880	7	46285940	A	T	-0.0338797	0.064302	0.00555197	0.000000001	0.000138124	36.18382911
57	rs112139215	7	73034559	A	C	0.0383734	0.068719	0.00535312	7.59976E-13	0.000188473	49.37592377
58	rs6462990	7	40913646	C	T	-0.0154022	0.411083	0.00275401	2.19999E-08	0.000114863	30.08945011
59	rs10499659	7	47274382	G	C	0.0260363	0.092994	0.00468276	2.69998E-08	0.000114355	29.95632459
60	rs10224503	7	107075569	C	A	0.0186439	0.237196	0.00318533	4.79999E-09	0.000125784	32.95062993
61	rs6467157	7	127660763	C	T	-0.0208345	0.295662	0.00301374	4.70002E-12	0.000180789	47.36271138
62	rs4141362	7	93159688	A	G	0.0176346	0.373165	0.00280157	3.09999E-10	0.000145484	38.11218024
63	rs2905323	7	22705723	C	T	-0.0190009	0.66245	0.00288838	4.79954E-11	0.000161462	42.2984962
64	rs798550	7	2760609	G	A	-0.0236319	0.296709	0.00295721	1.29987E-15	0.000233074	61.06319627
65	rs34776209	7	23513093	T	C	-0.0302654	0.247779	0.00313566	4.79954E-22	0.000341454	89.46771412
66	rs2003370	8	126468831	C	A	-0.0182835	0.208506	0.00333564	4.20001E-08	0.000110335	28.90333891
67	rs351776	8	28191306	C	A	0.0148469	0.547824	0.00271415	4.49997E-08	0.000109207	28.60769048
68	rs62496903	8	6446938	T	C	0.0353034	0.083882	0.00487313	4.30031E-13	0.00019155	50.18243097
69	rs2568232	8	38365841	G	T	0.0218703	0.795627	0.00338322	1E-10	0.000155551	40.74978411
70	rs72656010	8	57122215	C	T	-0.0258984	0.13182	0.00401205	1.09999E-10	0.000153521	40.21785182
71	rs10505073	8	106112262	C	G	0.0249536	0.182954	0.00351588	1.29987E-12	0.000186159	48.76979496
7	rs2608	8	12917	G	C	0.0197	0.3364	0.002	5.6001	0.0001	45.719

2	029		0126			706	08	86925	5E-12	74517	15476
73	rs12679821	8	142341543	T	C	0.0168897	0.481795	0.00271649	5.1E-10	0.000142442	37.3151218
74	rs7842144	8	23396874	G	T	-0.0172809	0.241046	0.00316294	4.70002E-08	0.000109264	28.6227127
75	rs13266210	8	41533514	G	A	-0.0255781	0.213786	0.00331512	1.20005E-14	0.000219931	57.61921808
76	rs768056	9	94266286	T	C	0.0201728	0.178061	0.00354178	0.000000012	0.000119116	31.20381788
77	rs1888190	9	113891000	T	C	0.0171806	0.55582	0.00272749	2.99999E-10	0.000145747	38.18109359
78	rs2159778	9	123678129	A	G	0.0167896	0.639076	0.00281608	2.5E-09	0.000130041	34.06596433
79	rs614274	9	125747038	T	G	-0.0313996	0.860444	0.00390546	8.99912E-16	0.000236782	62.03509318
80	rs7028020	9	38007747	G	A	-0.0153271	0.483541	0.00270424	1.40001E-08	0.000117333	30.73656528
81	rs145965565	9	98273305	G	T	0.0492291	0.09449	0.00464176	2.80027E-26	0.000414718	108.6721546
82	rs7854962	9	96900505	G	C	-0.023597	0.212965	0.00333293	1.39991E-12	0.000186658	48.90037352
83	rs28378473	9	139245460	C	T	-0.0288414	0.73377	0.00307424	6.4998E-21	0.000324997	85.15419065
84	rs4350272	10	25056118	G	A	-0.0231964	0.731337	0.003054	3.10027E-14	0.000211445	55.39539477
85	rs10883846	10	104958244	T	C	-0.0253	0.39359	0.00276847	6.29941E-20	0.000305549	80.05701809
86	rs9645500	10	70986723	G	T	0.0292466	0.691674	0.00291763	1.20005E-23	0.000364832	95.59519913
87	rs11187141	10	94467145	T	A	0.023367	0.37678	0.00279347	6.00067E-17	0.000256428	67.18336601
88	rs72814147	10	89605008	T	C	-0.0340651	0.05743	0.00579899	4.20001E-09	0.000125632	32.91103718
89	rs10821907	10	52648454	T	C	-0.0217875	0.179309	0.00356563	9.90011E-10	0.00013971	36.59927459
90	rs1801253	10	115805056	C	G	0.028157	0.73695	0.00308492	7.00003E-20	0.000307382	80.53744442
91	rs2901307	10	124128443	T	C	0.0192207	0.469451	0.00270872	1.29987E-12	0.000184028	48.21135499
92	rs5030317	11	32410337	G	C	-0.0190399	0.267635	0.00305177	4.39997E-10	0.000142112	37.22860613
93	rs10769199	11	46273240	G	T	-0.0150806	0.418834	0.00274666	0.00000004	0.000110716	29.00298476
94	rs10796820	11	69303727	T	G	-0.0172143	0.529237	0.00270795	2.1E-10	0.000147659	38.68215193
95	rs577044	11	111490420	T	G	0.0207626	0.714305	0.00298947	3.80014E-12	0.000175946	46.09368845
96	rs11222084	11	130273230	T	A	-0.0157805	0.369492	0.00279498	0.000000016	0.000116029	30.39504513

97	rs2508710	11	65229828	T	G	0.0204296	0.191068	0.00343499	2.69998E-09	0.000129018	33.79799787
98	rs72851023	11	2130620	T	C	0.0474408	0.0762	0.005154	3.40017E-20	0.00031686	83.02135323
99	rs4444073	11	10331664	C	A	-0.0154547	0.482626	0.00270353	1.09999E-08	0.00011928	31.2466539
100	rs10784514	12	66452879	T	C	0.0200027	0.681689	0.00290007	5.30029E-12	0.000173638	45.48894151
101	rs117202791	12	121519759	C	T	-0.0721901	0.012059	0.0124387	6.49995E-09	0.000124173	32.52870402
102	rs9669403	12	46798900	A	G	0.0163953	0.401996	0.00277663	3.50002E-09	0.000129239	33.85602185
103	rs76895963	12	4384844	G	T	0.0981802	0.02074	0.0104446	5.50047E-21	0.000391548	102.59824
104	rs118106744	12	21936398	T	C	-0.036509	0.05371	0.00597692	0.000000001	0.000135491	35.49387076
105	rs2291261	12	26875565	A	G	-0.0197307	0.487261	0.00270772	3.19963E-13	0.000194524	50.96156052
106	rs3184504	12	111884608	C	T	0.0266667	0.51828	0.00270064	5.40008E-23	0.000355081	93.03945416
107	rs7306710	12	66376091	C	T	-0.0387395	0.518954	0.00272361	6.4998E-46	0.000749296	196.4103047
108	rs7981781	13	28499962	A	G	-0.021103	0.220131	0.00327135	1.09999E-10	0.000152905	40.05649185
109	rs9603672	13	40648257	A	G	-0.0277665	0.887412	0.00430888	1.2E-10	0.00015406	40.35913341
110	rs61627123	13	73625868	C	T	-0.0245208	0.180093	0.00353325	3.90032E-12	0.000177566	46.51821682
111	rs12889267	14	21542766	G	A	-0.0229816	0.167024	0.00362265	2.19999E-10	0.000146961	38.49913387
112	rs72698731	14	101194339	C	T	-0.0260212	0.126249	0.00411916	2.69998E-10	0.000149383	39.13365334
113	rs7402983	15	99193276	C	A	-0.0251713	0.601548	0.00280338	2.70023E-19	0.00030373	79.5801506
1	rs1153	15	91428	T	C	0.0168	0.5264	0.002	5.6000	0.0001	36.925

14	9637		290			138	63	71191	3E-10	40956	80623
115	rs11631953	15	96862761	G	A	-0.0231727	0.241716	0.00319408	4.00037E-13	0.000196843	51.56930809
116	rs339969	15	60883281	A	C	0.0191689	0.615	0.00277728	5.10035E-12	0.000174004	45.58490386
117	rs36012	16	55730724	A	G	-0.0164336	0.437925	0.0027223	1.6E-09	0.00013295	34.82831098
118	rs74932341	16	67139348	T	C	-0.0422425	0.031926	0.00769377	0.00000004	0.000110302	28.89451677
119	rs9783782	16	88317285	G	T	0.0169297	0.308365	0.00293034	7.59994E-09	0.000122256	32.02645749
120	rs7205514	16	50271806	G	T	-0.0175738	0.756179	0.00316416	2.80001E-08	0.000113883	29.83264633
121	rs16952009	17	2102437	T	C	-0.0166026	0.348071	0.00284051	5.1E-09	0.000125098	32.77101722
122	rs7207516	17	79945083	A	G	0.0157419	0.663145	0.00286892	4.09996E-08	0.000110712	29.00207818
123	rs9909342	17	25652275	A	G	0.0213608	0.384088	0.0027938	2.09991E-14	0.000215881	56.55792096
124	rs222852	17	7140606	G	A	-0.0323462	0.430509	0.00272358	1.59993E-32	0.000513033	134.4478119
125	rs62070643	17	29166302	G	A	-0.0209738	0.269039	0.00305202	6.29941E-12	0.000173019	45.32669746
126	rs60839038	18	20711927	C	A	0.015827	0.473932	0.00275089	8.70001E-09	0.000124907	32.72085259
127	rs2551402	18	60990022	A	C	0.015025	0.480936	0.00271309	3.09999E-08	0.000112711	29.52577791
128	rs4798379	18	5618950	T	C	0.0176634	0.25155	0.00311586	1.40001E-08	0.0001748	30.77527802
129	rs2602708	19	4915341	A	G	-0.0188389	0.670413	0.00287224	5.40008E-11	0.000156839	41.0872373
13	rs8106744	19	40721877	C	G	-0.0187041	0.734144	0.00306302	0.000000001	0.000136562	35.77469192

0											
131	rs115955629	19	43596950	T	G	0.0253042	0.115397	0.00422653	2.1E-09	0.000130725	34.24523611
132	rs255773	19	54723546	T	C	-0.0188186	0.464661	0.0027436	6.89922E-12	0.000176185	46.15635342
133	rs817329	20	62597694	G	T	-0.016002	0.594201	0.0027618	6.90001E-09	0.000123487	32.34906666
134	rs2296528	20	57274151	C	G	0.0172851	0.631791	0.00279917	6.59994E-10	0.000139009	36.41558079
135	rs2423514	20	10693337	G	A	0.0186853	0.461917	0.00270977	5.40008E-12	0.000173557	45.46780487
136	rs11698914	20	31327144	G	C	-0.0288533	0.769702	0.00325305	7.29962E-19	0.000295144	77.32985591
137	rs6029174	20	39174880	G	T	0.0236637	0.383391	0.00282569	5.50047E-17	0.000264757	69.36610533
138	rs2229742	21	16339172	C	G	-0.0271141	0.103659	0.00443905	0.000000001	0.000136616	35.78864354
139	rs8139201	22	46443789	C	T	-0.0164346	0.332509	0.00287409	1.09999E-08	0.000119894	31.40757509
140	rs1801645	22	50356850	T	C	0.0174871	0.740654	0.00308667	0.000000015	0.000117479	30.7749089
141	rs5762980	22	29480905	G	A	-0.0192443	0.651026	0.0028351	1.10002E-11	0.000168277	44.08430196
142	rs41311445	22	42070374	C	A	-0.0333046	0.095638	0.00459962	4.49987E-13	0.000191872	50.26663469

Abbreviations: eaf=Effect allele frequency; se=standard error

Supplementary table 2: MR estimates of the effect of birth weight on outcomes.

Exposure	Outcome	MR						
		Method	nSNP	β	SE	p	OR	95%CI
Birth Weight	FVC	Inverse variance weighted	141	0.201939046	0.026374376	1.90864E-14	1.223773412	1.162-1.289
		MR Egger	141	0.206956938	0.080338712	0.011037101	1.229929608	1.051-1.44
		Weighted median	141	0.091387831	0.014837966	7.31809E-10	1.095693867	1.064-1.128
		Simple mode	141	0.086099803	0.03043001	0.005348819	1.089915099	1.027-1.157
		Weighted mode	141	0.067023455	0.025452054	0.009405887	1.069320558	1.017-1.124
	FEV1	Inverse variance weighted	141	0.170820698	0.02387067	8.30097E-13	1.186278028	1.132-1.243
		MR Egger	141	0.143138063	0.07267731	0.050883808	1.1538891	1.001-1.331
		Weighted median	141	0.081304442	0.014897769	4.8291E-08	1.084701075	1.053-1.117
		Simple mode	141	0.080197949	0.03858817	0.039507096	1.083501524	1.005-1.169
		Weighted mode	141	0.060377364	0.028326064	0.034793417	1.062237321	1.005-1.123
	FEV1/FVC	Inverse variance weighted	116	0.012060635	0.027020673	0.655345571	1.012133658	0.96-1.067
		MR Egger	116	-0.030482838	0.081227836	0.708152705	0.969977079	0.827-1.137
		Weighted median	116	0.015587161	0.039027954	0.689609882	1.015709275	0.941-1.096
		Simple mode	116	0.036920403	0.098300195	0.707914887	1.037610427	0.856-1.258
		Weighted mode	116	0.030903614	0.081452544	0.705085801	1.031386088	0.879-1.21
	lung volume	Inverse variance weighted	141	0.266674105	0.045816405	5.8674E-09	1.305614883	1.193-1.428
		MR Egger	141	0.365704808	0.139458001	0.009706849	1.441529652	1.097-1.895
		Weighted median	141	0.179905366	0.047952061	0.000175587	1.197104071	1.09-1.315
		Simple mode	141	0.111801058	0.139320142	0.423637862	1.118290363	0.851-1.469
		Weighted mode	141	0.042931396	0.104847762	0.682824888	1.043866279	0.85-1.282

Abbreviations: SE=standard error; OR=odds ratio; CI=confidence interval

Supplementary table 3: MR estimates of the effect of birth weight on mediators.

Exposure	Outcome	MR						
		Method	nSNP	β	SE	p	OR	95%CI
Birth Weight	p_Bacteroidetes	IVW	133	-0.173	0.080	0.030	0.841	0.719-0.983
	c_Bacteroidia	IVW	133	-0.173	0.080	0.030	0.841	0.719-0.984
	o_Bacteroidales	IVW	133	-0.173	0.080	0.030	0.841	0.719-0.984
	o_Pasteurellales	IVW	133	-0.330	0.126	0.009	0.719	0.562-0.92
	f_Pasteurellaceae	IVW	133	-0.329	0.126	0.009	0.719	0.562-0.92
	g_Blautia	IVW	133	0.194	0.075	0.010	1.215	1.048-1.408
	g_Subdoligranulum	IVW	133	0.166	0.080	0.038	1.181	1.009-1.381
	g_Bilophila	IVW	133	0.155	0.074	0.037	1.168	1.009-1.351
	g_Haemophilus	IVW	134	-0.303	0.126	0.016	0.739	0.577-0.945
	s_Subdoligranulum_unclassified	IVW	117	0.201	0.084	0.017	1.222	1.037-1.441
	s_Bilophila_unclassified	IVW	133	0.148	0.074	0.048	1.159	1.002-1.341
	s_Haemophilus_parainfluenzae	IVW	134	-0.301	0.126	0.017	0.74	0.578-0.948
	s_Bacteroides_plebeius	IVW	134	-0.440	0.154	0.004	0.644	0.476-0.87
	s_Ruminococcus_obeum	IVW	133	0.276	0.081	0.001	1.318	1.124-1.546
	s_Coprococcus_catus	IVW	133	0.181	0.091	0.045	1.199	1.004-1.431
Birth Weight	P23.PWY..reductive.TCA.cycle.I	IVW	133	0.304	0.154	0.0489	1.355	1.001-1.834
	P441.PWY..superpathway.of.N.acetylneuraminate.degradation	IVW	133	0.171	0.074	0.0207	1.187	1.027-1.372
	PRPP.PWY..superpathway.of.histidine..purine..and.pyrimidine.biosynthesis	IVW	133	0.165	0.075	0.0287	1.179	1.017-1.366
	PWY.5705..allantoin.degradation.to.glyoxylate.III	IVW	133	0.268	0.135	0.0481	1.307	1.002-1.704
	PWY.6612..superpathway.of.tetrahydrofolate.biosynthesis	IVW	133	0.185	0.094	0.0494	1.204	1.001-1.448
	PWY.7187..pyrimidine.deoxyribonucleotides.de.novo.biosynthesis.II	IVW	133	0.151	0.074	0.0408	1.163	1.006-1.343
	PWY.7196..superpathway.of.pyrimidine.ribonucleosides.salvage	IVW	133	0.145	0.074	0.0493	1.156	1.000-1.336
	PWY.7237..myo...chiro..and.scillo.inositol.degradati	IVW	133	0.184	0.072	0.0109	1.202	1.043-1.385

on PWY.7371..1.4.dihydroxy.6.naphthoate.biosynthesis.II	IVW	133	-0.162	0.076	0.0331	0.851	0.733-0.987

Abbreviations: SE=standard error; OR=odds ratio; CI=confidence interval

Supplementary table 4: MR estimates of the effect of mediators on birth weight.

Exposure	Outcome	MR						
		Method	nSNP	β	SE	p	OR	95%CI
p_Bacteroidetes	Birth Weight	IVW	13	0.013	0.011	0.258	1.013	0.991-1.035
c_Bacteroidia		IVW	13	0.013	0.011	0.258	1.013	0.991-1.035
o_Bacteroidales		IVW	13	0.013	0.011	0.257	1.013	0.991-1.035
o_Pasteurellales		IVW	3	-7.96E-05	0.014	0.995	1.000	0.973-1.027
f_Pasteurellaceae		IVW	3	-7.84E-05	0.014	0.995	1.000	0.973-1.027
g_Blautia		IVW	12	-0.005	0.014	0.732	0.995	0.968-1.023
g_Subdoligranulum		IVW	15	0.017	0.012	0.134	1.018	0.995-1.041
g_Bilophila		IVW	7	-0.009	0.014	0.516	0.991	0.964-1.018
g_Haemophilus		IVW	5	-0.007	0.009	0.446	0.993	0.975-1.011
s_Subdoligranulum_unclassified		IVW	14	0.020	0.012	0.114	1.020	0.995-1.045
s_Bilophila_unclassified		IVW	11	-0.003	0.011	0.789	0.997	0.977-1.018
s_Haemophilus_parainfluenzae		IVW	6	-0.004	0.013	0.739	0.996	0.972-1.021
s_Bacteroides_plebeius		IVW	7	0.010	0.009	0.280	1.010	0.992-1.029
s_Ruminococcus_obeum		IVW	13	0.010	0.008	0.240	1.010	0.993-1.027
s_Coprococcus_catus		IVW	7	-0.013	0.012	0.271	0.987	0.963-1.011
P23.PWY..reductive.TCA.cycle.I	Birth Weight	IVW	10	0.007	0.005	0.169	1.007	0.997-1.017
P441.PWY..superpathway.of.N.acetylneuraminate.degradation		IVW	12	3.31E-04	0.012	0.979	1.000	0.976-1.025
PRPP.PWY..superpathway.of.histidine..purine..and.pyrimidine.biosynthesis		IVW	15	0.028	0.011	0.010	1.028	1.007-1.05
PWY.5705..allantoin.degradation.to.glyoxylate.III		IVW	10	-0.005	0.008	0.572	0.995	0.979-1.012
PWY.6612..superpathway.of.tetrahydrofolate.biosynthesis		IVW	14	0.010	0.017	0.538	1.011	0.977-1.045
PWY.7187..pyrimidine.		IVW	9	-0.006	0.017	0.702	0.994	0.962-1.

deoxyribonucleotides.de.novo.biosynthesis.II							027
PWY.7196..superpathway.of.pyrimidine.ribonucleosides.salvage	IVW	7	-0.002	0.018	0.930	0.998	0.964-1.034
PWY.7237..myo...chiro..and.scillo.inositol.degradation	IVW	11	-8.99E-04	0.010	0.930	0.999	0.979-1.019
PWY.7371..1.4.dihydroxy.6.naphthoate.biosynthesis.II	IVW	9	0.006	0.015	0.675	1.006	0.978-1.036

Abbreviations: SE=standard error; OR=odds ratio; CI=confidence interval

Supplementary table 5: MR estimates of the effect of mediators on outcomes.

Exposure	Outcome	MR					
		Method	β	SE	p	OR	95%CI
p_Bacteroidetes	FVC	IVW	0.204	0.028	4.22E-13	1.227	1.161-1.297
c_Bacteroidia		IVW	0.204	0.028	4.23E-13	1.224	1.159-1.294
o_Bacteroidales		IVW	0.204	0.028	4.23E-13	1.229	1.163-1.299
o_Pasteurellales		IVW	0.202	0.028	6.52E-13	1.215	1.151-1.284
f_Pasteurellaceae		IVW	0.202	0.028	6.51E-13	1.228	1.162-1.298
g_Blautia		IVW	0.206	0.028	2.91E-13	1.224	1.159-1.294
g_Subdoligranulum		IVW	0.195	0.028	2.53E-12	1.227	1.161-1.297
g_Bilophila		IVW	0.205	0.028	2.98E-13	1.224	1.159-1.294
g_Haemophilus		IVW	0.202	0.028	5.77E-13	1.227	1.161-1.297
s_Subdoligranulum_unclassified		IVW	0.203	0.030	1.76E-11	1.225	1.154-1.299
s_Bilophila_unclassified		IVW	0.205	0.028	3.25E-13	1.227	1.161-1.297
s_Haemophilus_parainfluenzae		IVW	0.205	0.028	3.25E-13	1.227	1.161-1.297
s_Bacteroides_plebeius		IVW	0.201	0.028	1.13E-12	1.222	1.157-1.292
s_Ruminococcus_obeum		IVW	0.203	0.029	1.88E-12	1.226	1.158-1.297
s_Coprococcus_catus		IVW	0.208	0.028	1.83E-13	1.231	1.165-1.301
p_Bacteroidetes	FEV1	IVW	0.173	0.026	1.05E-11	1.189	1.131-1.25
c_Bacteroidia		IVW	0.173	0.026	1.05E-11	1.191	1.133-1.252
o_Bacteroidales		IVW	0.173	0.026	1.05E-11	1.194	1.136-1.255
o_Pasteurellales		IVW	0.175	0.025	6.73E-12	1.181	1.124-1.24
f_Pasteurellaceae		IVW	0.175	0.025	6.72E-12	1.188	1.13-1.249
g_Blautia		IVW	0.177	0.025	3.62E-12	1.191	1.133-1.251
g_Subdoligranulum		IVW	0.166	0.025	4.16E-11	1.189	1.131-1.25
g_Bilophila		IVW	0.172	0.025	1.35E-11	1.191	1.133-1.252
g_Haemophilus		IVW	0.174	0.025	6.40E-12	1.189	1.131-1.25
s_Subdoligranulum_unclassified		IVW	0.170	0.027	1.61E-10	1.186	1.125-1.249
s_Bilophila_unclassified		IVW	0.172	0.025	1.21E-11	1.188	1.13-1.249
s_Haemophilus_parainfluenzae		IVW	0.174	0.025	7.24E-12	1.190	1.132-1.251
s_Bacteroides_plebeius		IVW	0.169	0.025	3.60E-11	1.184	1.126-1.244
s_Ruminococcus_obeum		IVW	0.179	0.026	7.11E-12	1.196	1.136-1.258
s_Coprococcus_catus		IVW	0.182	0.025	7.90E-13	1.199	1.141-1.26
p_Bacteroidetes	Lung volume	IVW	0.267	0.048	2.99E-08	1.306	1.188-1.435
c_Bacteroidia		IVW	0.267	0.048	2.99E-08	1.290	1.174-1.417
o_Bacteroidales		IVW	0.267	0.048	2.99E-08	1.292	1.175-1.42
o_Pasteurellales		IVW	0.254	0.048	1.20E-07	1.293	1.177-1.421
f_Pasteurellaceae		IVW	0.254	0.048	1.20E-07	1.295	1.178-1.423
g_Blautia		IVW	0.256	0.048	1.16E-07	1.292	1.176-1.419
g_Subdoligranulum		IVW	0.257	0.048	8.83E-08	1.306	1.188-1.435
g_Bilophila		IVW	0.258	0.048	8.20E-08	1.290	1.174-1.417
g_Haemophilus		IVW	0.256	0.048	9.35E-08	1.306	1.188-1.435
s_Subdoligranulum_unclassified		IVW	0.259	0.051	3.88E-07	1.296	1.172-1.432
s_Bilophila_unclassified		IVW	0.256	0.048	1.01E-07	1.292	1.176-1.419
s_Haemophilus_parainfluenzae		IVW	0.256	0.048	9.42E-08	1.292	1.176-1.419
s_Bacteroides_plebeius		IVW	0.265	0.048	3.93E-08	1.303	1.186-1.432
s_Ruminococcus_obeum		IVW	0.259	0.049	1.48E-07	1.296	1.177-1.428
s_Coprococcus_catus		IVW	0.273	0.048	1.42E-08	1.313	1.195-1.443

Abbreviations: SE=standard error; OR=odds ratio; CI=confidence interval

Supplementary table 6. Heterogeneity and pleiotropy analysis of birth weight on outcomes

Exposure	Outcome	Heterogeneity						Pleiotropy	
		Method	Cochran's Q	P-value	Method	Cochran's Q	P-value	Egger-intercept (95%CI)	P-value
Birth weight	FVC	IVW	2030.396	0	MR-Egger	2030.460	0	0.002	0.947
Birth weight	FEV1	IVW	1486.506	4.08E-224	MR-Egger	1488.247	6.08E-224	0.002	0.687
Birth weight	FEV1/FVC	IVW	95.816	0.891	MR-Egger	96.125	0.899	0.002	0.580
Birth weight	Lung volume	IVW	366.297	2.24E-22	MR-Egger	367.788	2.29E-22	0.003	0.453

Abbreviations: IVW=inverse variance weighted

Supplementary table 7. Heterogeneity and pleiotropy analysis of birth weight on mediators.

Exposure	Outcome	Heterogeneity						Pleiotropy	
		Method	Cochran's Q	P-value	Method	Cochran's Q	P-value	Egger-intercept(95%CI)	P-value
Birth weight	p_Bacteroidetes	IVW	162.230	0.033	MR-Egger	165.101	0.027	-0.009	0.130
	c_Bacteroidia	IVW	162.198	0.033	MR-Egger	165.061	0.027	-0.009	0.131
	o_Bacteroidales	IVW	162.162	0.034	MR-Egger	165.021	0.027	-0.009	0.131
	o_Pasteurellales	IVW	129.896	0.511	MR-Egger	130.163	0.529	0.005	0.606
	f_Pasteurellaceae	IVW	129.890	0.511	MR-Egger	130.156	0.529	0.005	0.607
	g_Blautia	IVW	130.114	0.505	MR-Egger	130.545	0.519	0.004	0.513
	g_Subdoligranulum	IVW	166.943	0.019	MR-Egger	168.448	0.018	0.007	0.279
	g_Bilophila	IVW	109.133	0.918	MR-Egger	109.207	0.927	0.002	0.787
	g_Haemophilus	IVW	129.506	0.545	MR-Egger	129.740	0.564	0.005	0.629
	s_Subdoligranulum_unclassified	IVW	147.991	0.021	MR-Egger	149.508	0.020	0.007	0.280
	s_Bilophila_unclassified	IVW	113.024	0.870	MR-Egger	113.030	0.882	0.000	0.940
	s_Haemophilus_parainfluenzae	IVW	131.726	0.490	MR-Egger	132.057	0.507	0.006	0.566
	s_Bacteroides_plebeius	IVW	144.122	0.222	MR-Egger	144.157	0.240	-0.002	0.857
	s_Ruminococcus_obeum	IVW	116.440	0.814	MR-Egger	117.043	0.820	0.005	0.439
	s_Coprococcus_catus	IVW	124.204	0.650	MR-Egger	124.284	0.671	-0.002	0.777
Birth weight	P23.PWY..reductive.TCA.cycle.I	IVW	113.860	0.857	MR-Egger	114.415	0.863	-0.009	0.458
	P441.PWY..superpathway.of.N.acetylneuraminate.degradation	IVW	141.188	0.256	MR-Egger	141.228	0.276	0.001	0.847
	PRPP.PWY..superpathway.of.histidine..purine..and.pyrimidine.biosynthesis	IVW	144.335	0.201	MR-Egger	145.677	0.196	-0.006	0.272
	PWY.5705..allantoin.degradation.	IVW	124.261	0.649	MR-Egger	125.426	0.644	0.011	0.283

to.glyoxylate.III								
PWY.6612..superpathway.of.tetrahydrofolate.biosynthesis	IVW	153.109	0.091	MR-Egger	153.426	0.098	-0.004	0.603
PWY.7187..pyrimidine.deoxyribonucleotides.de.novo.biosynthesis.II	IVW	136.395	0.356	MR-Egger	139.666	0.307	0.010	0.079
PWY.7196..superpathway.of.pyrimidine.ribonucleosides.salvage	IVW	127.889	0.561	MR-Egger	129.410	0.548	-0.007	0.220
PWY.7237..myo...chiro..and.scillo.inositol.degradation	IVW	101.511	0.974	MR-Egger	101.569	0.977	-0.001	0.811
PWY.7371..1.4.dihydroxy.6.naphthoate.biosynthesis.II	IVW	104.276	0.959	MR-Egger	104.398	0.963	0.002	0.728

Abbreviations: IVW=inverse variance weighted

Supplementary table 8. Heterogeneity and pleiotropy analysis of mediators on birth weight.

Exposure	Outcome	Heterogeneity						Pleiotropy	
		Method	Cochran's Q	P-value	Method	Cochran's Q	P-value	Egger-intercept(95%CI)	P-value
p_Bacteroidetes	Birth weight	IVW	16.056	0.139	MR-Egger	16.819	0.157	-0.003	0.485
c_Bacteroidia		IVW	16.051	0.139	MR-Egger	16.817	0.157	-0.003	0.484
o_Bacteroidales		IVW	16.047	0.139	MR-Egger	16.816	0.157	-0.003	0.483
o_Pasteurellales		IVW	0.000	0.983	MR-Egger	2.701	0.259	0.024	0.348
f_Pasteurellaceae		IVW	0.000	0.985	MR-Egger	2.701	0.259	0.024	0.348
g_Blautia		IVW	20.253	0.027	MR-Egger	21.937	0.025	0.007	0.383
g_Subdoligranulum		IVW	20.903	0.075	MR-Egger	23.524	0.052	0.006	0.224
g_Bilophila		IVW	5.932	0.313	MR-Egger	7.245	0.299	0.006	0.341
g_Haemophilus		IVW	0.303	0.959	MR-Egger	4.010	0.405	0.026	0.150
s_Subdoligranulum_unclassified		IVW	18.445	0.103	MR-Egger	22.369	0.050	0.008	0.136
s_Bilophila_unclassified		IVW	8.063	0.528	MR-Egger	10.601	0.389	0.009	0.146
s_Haemophilus_parainfluenzae		IVW	3.041	0.551	MR-Egger	11.751	0.038	0.031	0.042
s_Bacteroides_plebeius		IVW	9.952	0.077	MR-Egger	13.211	0.040	0.010	0.257
s_Ruminococcus_obeum		IVW	8.627	0.656	MR-Egger	11.249	0.508	-0.006	0.134
s_Coprococcus_catus		IVW	7.352	0.196	MR-Egger	7.625	0.267	-0.003	0.685
P23.PWY..reductive.TCA.cycle.I	Birth weight	IVW	4.368	0.823	MR-Egger	4.385	0.884	-0.001	0.899
P441.PWY..superpathway.of.N.acetylneuraminate.degradation		IVW	13.195	0.213	MR-Egger	17.110	0.105	0.006	0.116
PRPP.PWY..superpathway.of.histidine..purine..and.pyrimidine.biosynthesis		IVW	19.348	0.113	MR-Egger	19.544	0.145	-0.002	0.723
PWY.5705..allantoin.degradation.to.g		IVW	18.110	0.020	MR-Egger	18.114	0.034	0.000	0.967

lyoxylate.III								
PWY.6612..superpathway.of.tetrahydrofolate.biosynthesis	IVW	62.463	0.000	MR-Egger	66.896	0.000	-0.010	0.374
PWY.7187..pyrimidine.deoxyribonucleotides.de.novo.biosynthesis.II	IVW	14.902	0.037	MR-Egger	15.426	0.051	-0.004	0.635
PWY.7196..superpathway.of.pyrimidine.ribonucleosides.salvage	IVW	11.838	0.037	MR-Egger	11.866	0.065	-0.001	0.918
PWY.7237..myo...chiro..and.scillo.inositol.degradation	IVW	8.792	0.457	MR-Egger	9.573	0.479	-0.004	0.400
PWY.7371..1.4.dihydroxy.6.naphthoate.biosynthesis.II	IVW	11.142	0.133	MR-Egger	14.618	0.067	0.009	0.183

Abbreviations: IVW=inverse variance weighted
